# Design considerations for optogenetic applications of soft micro-LED-based device systems across diverse nervous systems

**DOI:** 10.1016/j.bioactmat.2025.02.006

**Published:** 2025-02-19

**Authors:** Ju Young Lee, Taemin Kim, Shinil Cho, Jiho Shin, Woon-Hong Yeo, Tae Soo Kim, Ki Jun Yu

**Affiliations:** a Functional Bio-integrated Electronics and Energy Management Laboratory, School of Electrical and Electronic Engineering, Yonsei University, 50 Yonsei-ro, Seodaemungu, 03722, Seoul, Republic of Korea; b Department of Chemical Engineering, Texas A&M University, College Station, TX, 77843, USA; c George W. Woodruff School of Mechanical Engineering, Georgia Institute of Technology, Atlanta, GA, 30332, USA; d Wearable Intelligent Systems and Healthcare Center, Institute for Matter and Systems, Georgia Institute of Technology, Atlanta, GA, 30332, USA; e Wallace H. Coulter Department of Biomedical Engineering, Georgia Tech and Emory University School of Medicine, Atlanta, GA, 30332, USA; f Parker H. Petit Institute for Bioengineering and Biosciences, Georgia Institute of Technology, Atlanta, GA, 30332, USA; g YU-KIST Institute, School of Electrical and Electronic Engineering, Yonsei University, 50 Yonsei-ro, Seodaemungu, 03722, Seoul, Republic of Korea; h The Biotech Center, Pohang University of Science and Technology (POSTECH), 77 Cheongam-Ro, Nam-Gu, 37673, Pohang, Gyeongbuk, Republic of Korea

## Abstract

Optogenetics enables precise, cell-specific control of neural activity, surpassing traditional electrical stimulation methods that indiscriminately activate nearby cells, making it crucial for rehabilitation, neurological disorder treatment, and understanding neural circuits. Among light sources for delivering light to genetically modified cells, bio-implants integrated with Light Emitting Diodes (LEDs) have recently been the focus of extensive research due to their advantage of enabling local photogeneration. Unlike laser-based systems, which require tethered setups that hinder behavioral experiments, μ-LED-based devices allow for wireless operation, facilitating more natural movement in subjects. Furthermore, μ-LED arrays can be designed with higher spatial resolution compared to waveguide-coupled external light sources, enabling more precise control over neural activity. This paper presents design rules for implantable flexible optogenetic devices based on μ-LED, tailored to the unique anatomical and functional requirements of various regions of the nervous system. Integration of recent advancements in devices with μ-LEDs (e.g. wireless systems, optofluidic systems, multifunctionality, and closed-loop systems) enhances behavioral experiments and deepens understanding of complex neural functions in the brain, spinal cord, autonomic nervous system, and somatic nervous system. The combination of optogenetics with advanced bio-implantable devices offers promising avenues in medical science, providing more effective tools for neuromodulation research and clinical applications.

## Highlights

• Mechanisms of optogenetic neuromodulation and its application across various nervous systems are explained.
• Specific design rules for applying LED-based flexible devices to different nervous systems are overviewed.
• Recent studies in μ-LED-based devices for enhancing behavioral experiments and understanding neural functions are reviewed.
• The future directions for the development of bio-implantable devices for advanced neural modulation are discussed.

## Graphical abstract

**Figure:**
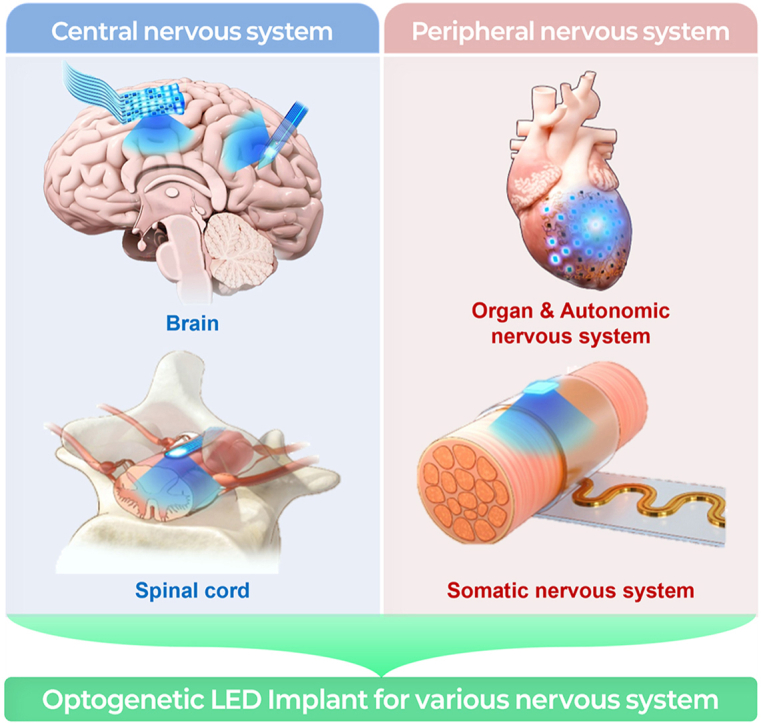
The figure illustrates the categorization of optogenetic LED implants across different nervous systems. These applications are grouped into the central nervous system (CNS), which includes the brain and spinal cord, and the peripheral nervous system (PNS), which is further divided into the organ & autonomic nervous system and the somatic nervous system. This classification highlights the diverse applications of optogenetic neuromodulation.

## 1 Introduction

The human body comprises various nervous systems, including the central nervous system (CNS) and peripheral nervous system (PNS) [[1], [2], [3]]. These organs and nervous systems interact closely to regulate physiological and behavioral functions [[4], [5], [6]]. Neuromodulation in these various nervous systems not only plays a crucial role in functional rehabilitation and addressing neurological disorders but also enhances our understanding of neural circuits, providing valuable insights into a wide range of human behaviors [5,7]. Traditionally, neuromodulation methods have included clinical drug therapy, which affects the entire body rather than targeting specific nerves or organs, leading to side effects and reduced precision [8,9]. In contrast, electrical stimulation allows for accurate targeting of specific neural pathways and real-time modulation of neural activity with millisecond precision [7,10]. However, it has the drawback of lacking cell specificity, as it stimulates all cells near the electrode equally, limiting its ability to target specific cell types [11,12].

To address these issues, optogenetic technology was developed two decades ago, which uses light to control the activity of neurons [[13], [14], [15]]. The optogenetic method involves introducing opsins, proteins that respond to specific wavelengths of light, into target locations using viral vectors [16]. When light is applied to these areas, it activates ion channels, enabling precise control of neuronal activity. This approach allows for highly specific targeting of neurons and pathways because light can be precisely controlled and directed to small, defined regions of tissue [17]. Additionally, different opsins can be expressed in distinct types of neurons, allowing for cell-type-specific stimulation [18]. With various genetic modifications, optogenetics enables both the activation and inhibition of neurons, providing versatility to perform neuromodulation in the desired manner [19].

Through the integration of optogenetic system with bio-implantable devices, researchers have enabled precise control of neural activity directly within the body, targeting specific neurons and pathways, including those affecting the CNS, and PNS [[20], [21], [22]]. When applying bio-implantable devices to various nervous systems, several considerations must be addressed. First, minimizing mechanical stress between the bio-implantable device and soft tissue is essential [23,24]. The mechanical mismatch between rigid bio-implantable devices and soft tissue can lead to friction or continuous irritation after implantation, causing damage to the surrounding tissue [25,26]. This can extend beyond tissue damage to trigger an inflammatory response and lead to the formation of fibrous encapsulation around the device, ultimately leading to malfunction [27]. A second consideration is the issue of device migration after implantation. If the device is not securely fixed in place within the target organ, it may move to an unintended location, preventing accurate stimulation of the target region [28]. To overcome this limitation, using soft, flexible, and small-sized devices that mimic soft tissue is essential, as this reduces mechanical mismatch while promoting accurate, stable, and long-term operation [25,[29], [30], [31], [32]]. The application of a soft, flexible, and small-sized form factor not only reduces the potential for migration, allowing the device to be more easily stabilized in position, but also minimizes tissue damage during implantation and lowers the risk of immune response [33]. In addition to reducing thickness and modulus, various bioinspired adhesive surfaces have been developed to prevent device migration. Given the body's internal environment, achieving effective wet adhesion is particularly important for implants [[34], [35], [36]]. Chen et al. have categorized different bioinspired wet adhesive surfaces based on their adhesion mechanisms, providing a comprehensive overview of current advancements in this field [37]. Moreover, through the integration of biocompatible materials, these devices minimize side effects (e.g. inflammation and foreign body response) and ensure long-term stable operation, providing a significant solution for long-term disease management [[38], [39], [40]]. These advancements bring innovation to the medical field, opening new possibilities for treating various neurological and organ-related disorders, while representing a roadmap to new therapeutic options [[41], [42], [43], [44]].

Efficient delivery of light to the target site is another fundamental requirement in optogenetic applications, as both the light source and its propagation pathway must be carefully designed to maximize efficacy. Generally, optogenetic stimulation relies on two types of light sources: coherent light (lasers) and incoherent light (μ-LEDs) [45]. Lasers provide high-intensity, collimated beams with minimal divergence, enabling precise targeting of deep neural structures with minimal scattering losses [46,47]. However, laser-coupled systems suffer from limitations in terms of device miniaturization and portability, as they typically require tethered optical fibers for light delivery, which in turn constrains behavioral experiments in freely moving animals [48]. Additionally, their high power consumption and the need for external alignment components make them less favorable for fully implantable and long-term studies [49]. In contrast, μ-LEDs offer distinct advantages, including compact size, lower power consumption, and the ability to integrate directly into untethered, wireless systems. These attributes make μ-LEDs particularly well-suited for applications involving behavioral experiments in freely moving animals, where unrestricted movement and long-term stability are crucial [[50], [51], [52], [53]].

To deliver μ-LED-generated light to the target region, two primary approaches are employed: waveguide-assisted delivery and direct integration at the stimulation site. Waveguides provide controlled light propagation, enabling precise spatial control while minimizing off-target illumination [54,55]. They also facilitate integration with microelectrode arrays, supporting simultaneous optical stimulation and neural recording [[56], [57], [58]]. However, waveguides introduce power loss due to coupling inefficiencies and require careful alignment, adding complexity to device fabrication and implantation [20]. Alternatively, directly interfacing μ-LEDs with the target site eliminates the need for optical pathways, minimizing power loss while maximizing spatial resolution. High spatial resolution is particularly important in applications requiring precise neural circuit mapping, selective activation of specific neuronal populations, and targeted stimulation of small functional regions within the nervous system [[59], [60], [61]]. These direct-contact μ-LED arrays enable targeted stimulation of small neuronal ensembles while reducing unintended activation of adjacent pathways, which is crucial for studying fine motor control, sensory processing, and complex decision-making circuits. This strategy has been successfully implemented in diverse tissue environments, including the central nervous system, peripheral nerves, and even cardiac or muscle interfaces, expanding the scope of optogenetic modulation beyond traditional deep-brain applications.

Since the various nervous systems widely distributed throughout the body have distinct characteristics in terms of morphology and physical properties, bio-implantable devices must be designed with consideration of their unique anatomy. Thus, in this paper we explain how these devices are designed with different form factors and specific characteristics to enhance behavioral experiments and contribute to a deeper understanding of complex neural functions. Our article consists of three parts: 1) Introduction of the process of optogenetics, the advantages of this technique, and characteristics of various channels used for neuromodulation, 2) Design rules of bio-implantable μ-LED device on various nervous systems, organs and their recent advancements, 3) The future perspective of bio-implantable μ-LED device.

**Fig. 1:**
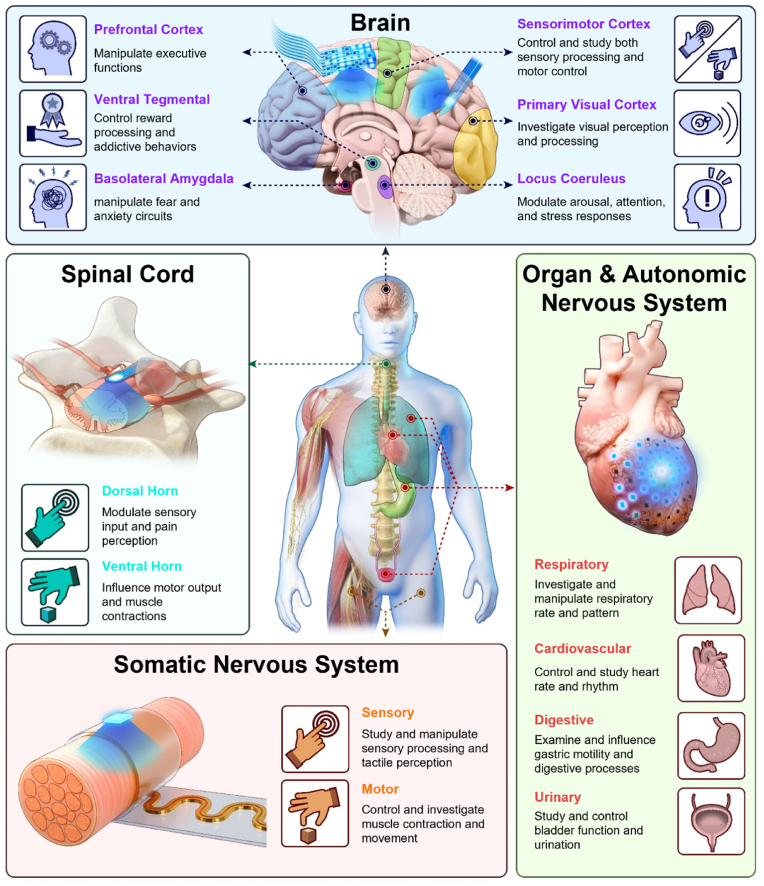
Illustration of various neuromodulation functions using bio-implantable devices combined with μ-LED for optogenetic stimulation across the nervous system. The nervous system is divided into the central nervous system (brain and spinal cord), organs and the peripheral nervous system (autonomic and somatic nervous system), with bio-implantable devices targeting specific regions for neuromodulation.

Fig. 1 illustrates the various neuromodulation functions within the nervous system, which is broadly categorized into the CNS (brain and spinal cord), organs and the PNS (autonomic nervous system (ANS) and somatic nervous system (SNS)) [62]. In each area, optogenetic neuromodulation techniques are employed to regulate different neural activities. In the brain, specific regions are associated with various functions, and devices are either attached to the surface or implanted into deeper region of the brain (e.g. intracortical and subcortical area) to target these regions for neuromodulation. Optogenetic neuromodulation in the brain spans a wide range of functions across different areas, such as the prefrontal cortex (manipulating executive functions) [63], ventral tegmental (VTA) area (controlling reward processing and addictive behaviors) [64], basolateral amygdala (modulating fear and anxiety circuits) [65], sensorimotor cortex (controlling sensory and motor processing) [66,67], primary visual cortex (visual perception) [68], and locus coeruleus (regulating arousal, attention, and stress) [69]. In the spinal cord, devices are often inserted into the epidural space, located beneath the spinal bones and dura mater. Optogenetic neuromodulation in the spinal cord is broadly categorized into two regions: the dorsal horn, which modulates sensory input and pain perception, and the ventral horn, which controls motor output and muscle contractions [[70], [71], [72]]. Devices applied for optogenetic neuromodulation in various organs are either directly attached to organs or attached on autonomic nerves that regulate organ function. These organs regulate essential bodily functions, such as the lungs (manipulating respiratory rate and pattern) [73], heart (controlling heart rate and rhythm) [74], stomach (regulating gastric motility and digestive processes) [75], and bladder (controlling bladder function and urination) [76]. In the SNS, devices are designed to wrap around nerves, receiving signals from the skin to facilitate sensory processing and tactile perception or transmitting signals to muscles to control contraction and movement [77,78]. While optical neuromodulation offers a versatile approach to studying neural functions across various regions of the nervous system, achieving comprehensive coverage and precision remains challenging. This limitation necessitates a more tailored approach in device design, as a single-form device cannot effectively address the diverse anatomical and functional requirements of these regions. Each region of the nervous system has unique characteristics that demand specific design considerations to ensure accurate modulation and measurement.

Following this, we investigate the development of bio-implantable devices with integrated μ-LEDs for optogenetic stimulation, highlighting how these advancements extend beyond merely incorporating light sources for neuromodulation [79]. These devices have been engineered to overcome a range of limitations by incorporating additional functionalities. For instance, they address the challenges of tethered devices in in vivo experiments through wireless operation [51,80], solve the lifespan issues of battery-dependent systems by adopting battery-free designs [81,82], and combine optogenetic stimulation with drug delivery in optofluidic systems for more comprehensive therapies [83,84]. Furthermore, they enable real-time diagnostics and treatment through closed-loop multifunctional capabilities [85,86] and ensure long-term stable operation with fully implantable systems [[87], [88], [89]]. Through these innovations, bio-implantable devices are advancing the field of neuromodulation, offering more effective and versatile tools for the study and treatment of complex neural circuits.

## 2 Optogenetic technology

**Fig. 2:**
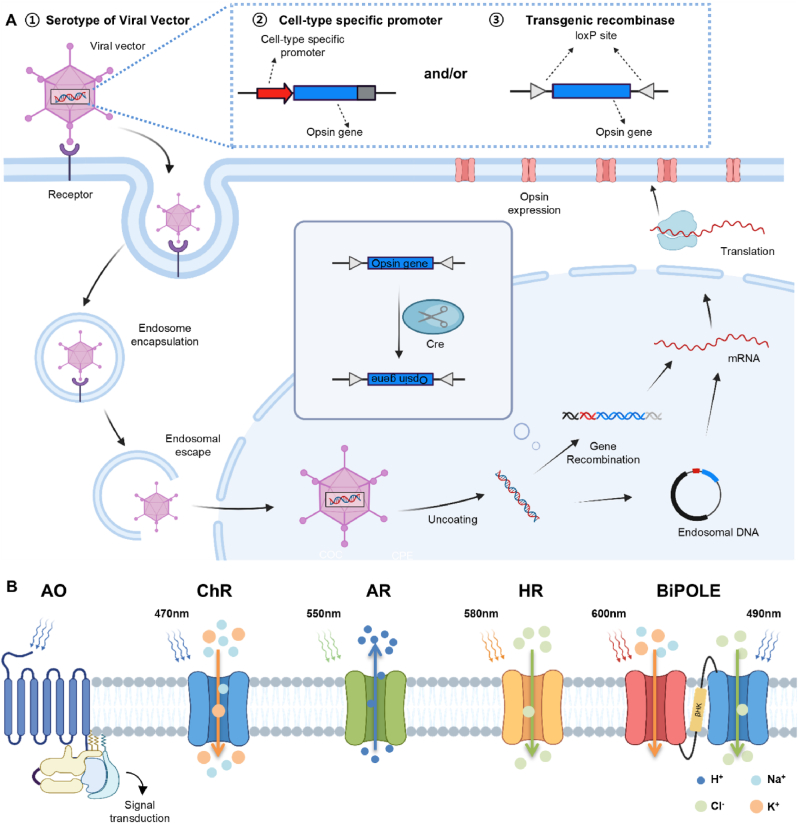
Overview of optogenetic technology. (A) MO gene delivery mechanism via viral vector. Serotype of viral vector, cell-type specific promoter, and Cre-loxP system contributes to cell-type specific expression of MO on target cell type. Inset: Cre recognizes loxP sites of delivered DNA and recombine into host cell DNA. Created in BioRender. Lee, J. (2025) https://BioRender.com/i62z511. (B) Types of opsins, animal opsin (AO), microbial opsins (Channelrhodopsin (ChR), Archaeorhodopsin (AR), Halorhodopsin (HR)), engineered opsin (BiPOLE). Created in BioRender. Lee, J. (2025) https://BioRender.com/p27s692.

Compared to conventional electrical stimulation methods, a major advantage of optogenetics is in its ability to target and stimulate specific cell types [90]. This precision is achieved by delivering opsin-expressing genes specifically to the desired cell type via viral vectors (Fig. 2A). (1) The tropism of viral vectors is one determinant of their specificity [91], which is closely related to the serotype of the viral vector. By appropriately modifying the surface proteins of the viral vector, it is possible to enhance interactions with specific host cell receptors, thereby increasing the efficiency of infection. The viral vectors are then introduced into the cell nucleus through series of steps including endosome encapsulation and endosomal escape. Another predominant approach is employed in the following step to compensate for the insufficient cell-specificity of viral serotypes, the strategic modification of delivered sequences, which includes the use of (2) cell type-specific promoters [92] and (3) transgenic recombinases [93]. A cell type-specific promoter is co-delivered with the opsin gene, positioned upstream to facilitate opsin gene transcription exclusively in the target cells. While the cell-type specific promoters are involved in the transcriptional regulation of genes, transgenic recombinases interfere in a preceding step, the gene recombination phase. A prominent example of utilizing transgenic recombinase is the Cre-loxP system [91,94], in which Cre recombinase recognizes loxP sites and facilitates the site-specific recombination of the opsin gene at a desired location within the genome. These approaches are not mutually exclusive and can be used together to achieve higher specificity [95].

Opsins can be broadly categorized into animal opsins and microbial opsins (MOs) (Fig. 2B). Animal opsins are classified as G-protein-coupled receptors (GPCRs) [96], which transmit signals to cells through a series of downstream signaling events. This indirect and complex signaling pathway makes it difficult to achieve immediate and precise cellular responses [97], complicating their application in optogenetics. In contrast, MOs function as direct ion channels or pumps, allowing ions to flow across the membrane immediately upon light activation, thus providing rapid and precise temporal control over neuronal activity [98]. Additionally, while animal opsins have long and complex sequences, making them difficult to manipulate [78], MOs are highly amenable to genetic manipulation due to its simple and short gene sequence. These characteristics of MOs allows researchers to create variants with tailored properties, enhancing ion selectivity, light sensitivity, and kinetic profile.

Different MOs have distinct characteristics, such as activation wavelength, transporting ions, light intensity required for an activation, and kinetics (reaction time, deactivation time, and recovery time). Therefore, it is important to choose the appropriate opsin when designing optogenetic devices and planning in vivo experiments. Table 1 highlights how different opsins are specifically tailored for activation or inhibition in various target organs, emphasizing their application in achieving precise control over neural activity. The most fundamental application of optogenetics will be the activation of neurons. Channelrhodopsin (ChR), originated from green algae, is the first and foremost opsin subfamily to be utilized for neural activation, as Boyden et al. introduced channelrhodopsin-2 (ChR2) into mammal neurons for optogenetic modulation [14]. ChR2 basically reacts to external blue light(maximum at 470 nm) and work as an inwardly rectifying non-selective cation channel(H^+^, Na^+^, K^+^, Ca^2+^ in order of larger permeability) [99], thus depolarizing the cell.

**Table 1:** Opsins for optogenetics: Types and applications in various organs.

Family	Name	Peak wavelength (nm)	Ions	Activation /Inhibition	Applied Organs	Characteristics
Channelrhodopsin	ChR2 [14]	473	H+, Na+, K+, Ca2+	activation	Motor cortex [140], Somatosensory Cortex [119], Spinal cord [216], Stomach [234], Sciatic nerve [142,252]	–
	ChR2 (H134R) [107]	450		activation	Brain VTA [173,180], Spinal cord [53,120], Sciatic nerve [146]	Faster kinetics than ChR2
	ChETA [108]	490		activation	Bladder [230]	ultrafast kinetics
	Chrimson [105]	590		activation	Motor cortex [179], Spinal Cord [53]	Red-shifted ChR
	ChrimsonR [105]	590		activation	Brain VTA [126,180], VTA-6VV pathway [182]	Faster off-kinetics compared to Chrimson
	ChRmine [106]	585		activation	–	High light sensitivity
	stGtACR2 [258]	470	Cl-	inhibition	Brain VTA [126]	Inhibitory channelrhodopsin with prolonged open state
Halorhodopsin	NpHR [102]	593	Cl-	inhibition	–	–
Archaerhodopsin	AR3(Arch) [103]	566	H+	inhibition	Bladder [101]	–
	ArchT [259]	532	H+	inhibition	Visual cortex [60], Heart [227]	3-fold higher light sensitivity than Arch

Halorhodopsin (HR) and Archaeorhodopsin (AR) are frequently utilized tools for silencing neuronal activities [100,101], though they achieve this through different mechanisms. NpHR, commonly used HR derived from Natronomonas pharaonic, functions by pumping anions (Cl^−^) into the cell in response to yellow light (peak activation at 580 nm) [102]. This influx of chloride ions leads to the hyperpolarization of the target neuron, effectively inhibiting its activity. Conversely, AR operates as a proton pump, expelling intracellular protons (H^+^) when exposed to green light [103]. This outward flow of protons similarly results in hyperpolarization, thereby silencing neuronal activity. Despite their distinct mechanisms—chloride ion influx for HR and proton efflux for AR—both opsins ultimately achieve neuronal inhibition.

The previously mentioned opsins, while effective in certain contexts, are not optimal in some aspects. For instance, ChR2 has relatively slow off-kinetics, with closing rate up to 13.5 ms [104], which can hinder temporal precision in high-frequency stimulation applications. Additionally, the blue light required for ChR2 activation has a relatively shallow tissue penetration depth, making it less suitable for targeting deeper regions in in vivo experiments. To compensate with some drawbacks of conventionally used MOs, there have been many trials to disinter various opsins to improve the effectiveness of optogenetics. These enhancements primarily focus on improving speed, light sensitivity, spectral response range, expression levels, and reducing cytotoxicity [[105], [106], [107], [108], [109]].

Efforts to obtain optimized opsins generally follow two main approaches: screening a wider range of natural MOs to find more optimized opsins, or genetically engineering opsins. One prominent example of MO screening is found in the development of red-shifted opsins [105,106]. Red-shifted opsins use longer wavelengths of light, allowing for deeper tissue stimulation and reducing phototoxicity compared to blue light, thereby minimizing the deterioration of the experimental subject's condition. Chrimson is one of the most used naturally occurring red-shifted opsins, responding to wavelengths near 590 nm and offering fast kinetics, making it suitable for temporally precise experiments [105]. However, it exhibits suboptimal performance in terms of photocurrent generation. In contrast, recently discovered ChRmine shows superior photocurrent and light sensitivity [106], but its slower closing kinetics present a trade-off [110]. The contrast between the two naturally occurring opsins highlights the need to compromise one advantage for another, depending on the experimental requirements.

A genetic approach provides an alternative strategy for optimizing microbial opsins, enabling modifications that enhance their utility in optogenetics. For instance, point mutations that substitute specific amino acids in the opsin sequence can transform naturally occurring microbial opsins into more user-friendly tools for optogenetic applications. ChR2 H134R exhibits increased light sensitivity compared to wild-type ChR2 [107], while ChETA [108] has been modified to display faster kinetics. Similarly, the ChR2 C128 variant demonstrates a prolonged open-state relative to the original ChR2 [111]. Additionally, ChrimsonR, a K176R mutant of Chrimson, has been engineered to red-shift its responsive wavelength, allowing spike generation at wavelengths as long as 735 nm [109]. Beyond simple point mutations, it is also possible to apply more advanced manipulation of opsin gene sequence that allow for more precise control of neurons. Bidirectional manipulation of neurons – activating and silencing neurons – has been a challenge that engineers strive to address. Bidirectional control is possible by using excitatory and inhibitory opsins with non-overlapping response wavelengths simultaneously. However, simply injecting two MOs typically results in an imbalance in stoichiometric expression, making precise co-location of opsins difficult. Thus, gene fusion methods, such as deploying a 2A peptide sequence [112] or combining two distinct opsin genes in a single tandem expression cassette [113], have been developed. A two-channel fusion protein called BiPOLE, which fuses GtACR2 and Chrimson (max response at 470 nm and 590 nm, respectively), allows bidirectional control depending on the wavelength of light illuminated [114]. Appropriately selecting and incorporating such genetic engineering approaches with novel LED device will reveal previously unknown neural responses, thus broadening understanding of neuroscience.

## 3 Implantable device for optogenetic modulation

Implantable LED devices for optogenetics have garnered significant attention due to their ability to provide precise, localized stimulation of neural circuits with high temporal resolution. The primary design considerations for these devices involve mechanical, optical, and thermal aspects to ensure compatibility and functionality within the biological environment.

From a mechanical standpoint, the devices must be flexible and conformable to match the soft, complex, and dynamic nature of biological tissues [115,116]. This includes employing materials that exhibit mechanical properties similar to those of human tissues, such as low modulus and high stretchability, to minimize irritation and maintain long-term stability [117]. Shape-memory materials [118] and stretchable substrates [119,120] have been developed to enhance conformability and integration with target tissues. Additionally, the miniaturization of the devices is crucial to minimize invasiveness and avoid damage to the surrounding tissues during and after implantation [49,121].

Optically, the devices need to ensure efficient light delivery to target cells with minimal loss [90,122]. The emission wavelength must match the absorption spectrum of the opsins used in optogenetic applications [123,124], with the device offering sufficient power density to penetrate biological tissues without causing thermal damage [90,125]. Multichannel and dual-color LEDs have been integrated to enable precise spatiotemporal control over neural activation and inhibition, thereby increasing the versatility of optogenetic applications [[126], [127], [128], [129]]. Furthermore, the devices should be designed to emit light uniformly to ensure controlled and reproducible light propagation. Maintaining a well-defined spatial light profile is crucial for consistent optogenetic stimulation, as variations in illumination intensity can lead to differences in neuronal activation [90].

Thermal management is another critical consideration, as excessive heat generated by the LEDs can lead to tissue damage and inflammation [122,130,131]. The device should operate within a safe thermal range to avoid inducing thermal lesions while delivering sufficient light intensity for effective optogenetic modulation [132,133]. Integration of thermal sensors and feedback mechanisms within the device can help in real-time monitoring and regulation of temperature during operation [60,134,135].

Additionally, the device should incorporate wireless power and data transmission to eliminate physical tethers that could restrict the subject's natural movements and interfere with long-term experiments [[136], [137], [138]]. Wireless power delivery for in vivo optogenetics can be divided into battery-powered and fully implantable, battery-free methods. Battery-powered systems often use rechargeable batteries with inductive coupling or photovoltaic for wireless charging [[139], [140], [141]]. Fully implantable systems, on the other hand, rely on methods like inductive power transfer, radio frequency (RF) energy harvesting, photonic power delivery, and triboelectric power delivery to wirelessly power the device without a battery [52,87,[142], [143], [144], [145]]. Data transmission is often achieved using various wireless communication methods, including Bluetooth Low Energy (BLE), Near Field Communication (NFC), RF antennas, and optical methods, allowing for flexible, real-time communication and wireless monitoring without physical tethers [53,101,126,139,140,[146], [147], [148], [149]]. Recent advancements now allow simultaneous wireless recording of bio-signals, such as electrocorticography (ECoG) [150,151], electromyography (EMG) [53,101], and electrocardiography (ECG) [152,153], providing deeper insights into the physiological responses to optogenetic stimulation [[154], [155], [156]]. This integration facilitates closed-loop experimental designs, where neural activity can dynamically modulate stimulation parameters in real time, paving the way for more sophisticated and physiologically relevant studies.

Although general design rules guide the development of implantable LED devices for optogenetics, these devices must be specifically tailored to the nervous system they target. Table 2, Table 3 summarize the design rules and thermal management strategies for optogenetic applications in the CNS and PNS, highlighting key features tailored to specific neural targets. The brain, spinal cord, ANS, and SNS each present unique anatomical and functional challenges that necessitate distinct design considerations for effective and safe neural stimulation.

**Table 2:** Design rules and thermal management strategies for optogenetic applications in the central nervous system.

Target	Purpose	Key Feature	Optical Power Denstiy	Temperature	Illumination Protocol	Reference
Secondary Motor cortex	Locomotion behaviors	Wireless solar powered battery charging system	<40 mW/mm2	<1 °C	20Hz pulse, 10 % duty cycle	[140]
striatum	Develop a fully implantable, wireless optogenetic system	Fully implantable, battery free system	15.15 mW/mm2	<1 °C	25 % duty cycle	[87]
Ventral Tegmental Area (VTA)	Social behavior	Colocalized, bidirectional optogenetic system	1) 35 mW/mm2 at 630 nm 2) 40 mW/mm2 at 480 nm	<1 °C	1) 20Hz·10 ms pulse, 20 % duty cycle at 630 nm 2) continuous operation 480 nm	[126]
Visual cortex (V1)	Two-alternative forced-choice luminance discrimination task	5x5 LED array for larger cortical area	1 LED with 96 mJ	<1 °C	1/4Hz·2s pulse, 50 % duty cycle	[60]
primary & secondary motor cortex	Jaw, wrist, neck, and whisker movement	Vertical LED using anisotropic conductive film for red light emissions	16mW/mm2	<0.5 °C	10Hz·10 ms pulse, 10 % duty cycle	[179]
Ventral (6VV) and dorsal (6VD) regions of area 6V & Ventrolateral prefrontal cortex (vlPFC)	Risk-return decision	29 LED array with 18 electrodes for simultaneous ECoG recordings and optogenetics	1) 3.13mW/mm2, 2) 16mW/mm2	<0.5 °C	10Hz·20 ms pulse, 20 % duty cycle	[182]
dorsal horn epidural space	Nocifensive behavior	Fully implantable, soft & stretchable miniaturized system	10mW/mm2	<0.1 °C	20Hz·10 ms, 20 % duty cycle	[120]

**Table 3:** Design rules and thermal management strategies for optogenetic applications in the peripheral nervous system.

Target	Purpose	Key Feature	Optical Power Denstiy	Temperature	Illumination Protocol	Reference
Heart	Pacemaking, strain sensing	Negative gauge factor strain sensor, closed-loop system	75∼90mW/mm2	N/A	40 % at diastole, 13 % at systole	[227]
Bladder	Micturition control, continuous bladder monitoring	Multifunctional sensor (Strain gauge, EMG electrodes, Temp meter)	2.4mW/mm2	0.2 °C	pulse duration 5s	[230]
Stomach (vagus nerve)	Apetite suppression	Organ-innervating nerve targeting device	10mW/mm2	0.2 °C	5 ms pulse, 10 % duty cycle	[234]
Sciatic nerve	Noniceptor activation (protective pain behaviour and inflammation)	Nerve cuff fixed with sutures passing through anchoring holes, subcutaneous connector (LED to head-mounted controller)	91mW/mm2	<0.5 °C	0.1–30Hz·1–50 ms pulse, 2 % duty cycle	[146]
Sciatic nerve	Noniceptor activation (aversive behaviour)	Nerve cuff made of soft elestomer to naturally curl around the nerve, fully implanted, wireless simultaneous pharmacological and optogenetic neuromodulation	N/A	<1 °C	1) 1Hz·10 ms pulse, 1 % duty cycle 2) 10Hz·10 ms pulse, 10 % duty cycle	[142]
Sciatic nerve	Motor neuron activation (muscle contraction)	Ultrathin OLED device with 2um thickness, MRI, fMRI compatible	0.5mW/mm2	<0.3 °C	2Hz·5 ms pulse, 1 % duty cycle	[252]
Sciatic nerve	Motor neuron activation (hindlimb movements)	Shape-memory spiral desing to self-wrap aroung the nerve, multisite stimulation for fascicle-specific stimulation	3.2mW/mm2	<0.5 °C	2Hz·5 ms pulse, 5 % duty cycle	[118]

### 3.1 Central nervous system (CNS)

#### 3.1.1 Brain

The brain, with its complex anatomy and diverse functional regions, presents unique challenges for the application of bio-implantable device. It comprises various structure, from the cortical surface to deeper subcortical areas, each playing critical roles in executive control, emotional regulation, sensorimotor processing, and addictive behaviors [157,158]. Bio-implantable devices for the brain need to precisely target specific regions with minimal damage to the surrounding brain tissue [159]. Broadly, devices applied to the brain can be categorized into two types: 1) inserted into deeper brain regions for more direct and localized interventions and 2) implanted on the brain surface for non-invasive or minimally invasive modulation. In the following sections, we will discuss the detailed design rules for each of these categories.

##### 3.1.1.1 Neural probe

Neural probes are devices designed to be implanted in the brain by removing the cranium and inserting the probe into a specific intracranial region to measure neural signals or to deliver precise neuromodulation at targeted sites [160]. Since these devices are directly implanted into the brain, it is crucial to minimize brain cell damage caused by the insertion. Therefore, minimizing the device's overall dimensions while simultaneously reducing the mechanical mismatch with brain tissue is key to designing the device's structure, which is particularly essential for implanted probes to minimize potential damage [161,162]. However, securing sufficient bending stiffness to ensure that the device can be inserted into the correct position without bending is also vital for accurate brain targeting [163]. To achieve this balance, establishing an additional insertion process protocol is important. For this, techniques like using an insertion shuttle or coating the neural probe with bioresorbable materials like polyethylene glycol (PEG) can provide sufficient bending stiffness during insertion while later dissolving to enable flexibility after implantation [[164], [165], [166], [167]].

**Fig. 3:**
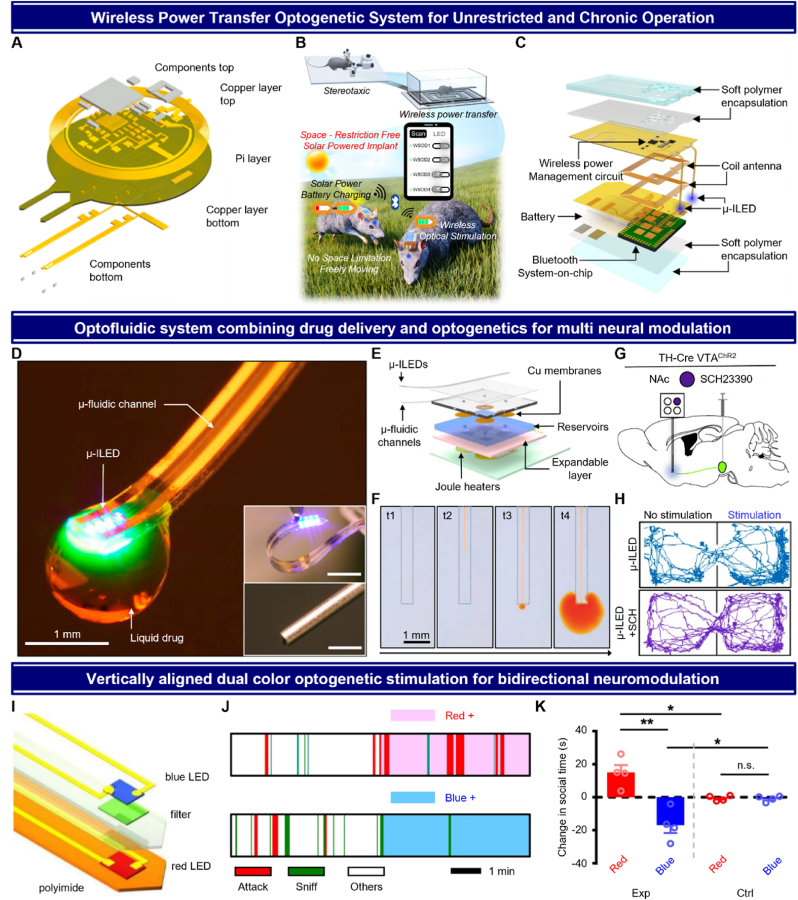
Various advanced optogenetic devices on deep brain regions. (A) A fully implantable, battery-free wireless optogenetic system utilizing magnetic resonant coupling at 13.56 MHz, enabling angle- and position-independent power transfer. The absence of a battery minimizes weight and reduces immune response risks. Reproduced from Ref. [87], with permission from Springer Nature. (B) A solar-powered wireless optogenetic system enabling space restriction-free and chronic behavior experiments. The system charges via solar energy and provides precise neural stimulation through mobile-controlled LEDs. From Ref. [140]. Reprinted with permission from AAAS. (C) A fully implantable optogenetic system combining battery-free and battery-powered advantages. It uses inductive power transfer for wireless recharging, enabling reliable and continuous operation for chronic studies. Reproduced from Ref. [139], with permission from Springer Nature. (D) Optofluidic device with integrated μ-LEDs and μ-fluidic channels. The inset compares a flexible device with a conventional rigid metal cannula. The μ-fluidic outlet and μ-LED are designed to target the same region, allowing for simultaneous optical stimulation and precise drug delivery to the specific neural area. (E) Schematic diagram of the optofluidic system, highlighting the combined μ-LED and drug delivery system, enabling precise control over both light and chemical stimulation. The μ-fluidic channel is operated via a Joule heater, providing controlled release of drugs in tandem with optical stimulation. (F) Time-sequenced delivery of dyed liquid through the microfluidic channel, illustrating the precise temporal control over fluid flow. The system demonstrates the capability to release liquids, ensuring targeted and programmable delivery for real-time modulation. (G) In vivo schematic of simultaneous optogenetic and pharmacological stimulation. The device is implanted in the NAc region, delivering both optical stimulation via a μ-LED and SCH23390 via microfluidic channels. (H) Behavioral results from real-time place testing, comparing movement trajectories in mice with or without μ-LED stimulation and SCH drug delivery. The experiment demonstrates simultaneous optogenetic and pharmacological modulation, with clear distinctions in movement patterns observed under combined stimulation, confirming real-time interaction between the two modalities. (D to H) Reprinted from Ref. [173], Copyright (2015), with permission from Elsevier. (I) Exploded view of a vertically aligned dual color optogenetic control device, demonstrating its design with a red μ-LED, optical filter, and blue LED stacked for bidirectional neuromodulation. The introduction of the optical filter allows efficient use of both wavelengths, enabling precise and effective control of neural activity. (J) Behavioral sequence data from social interaction tests using the dual color optogenetic device, color-coded to distinguish attack, sniffing, and other behaviors represent like locomotion and resting. (K) The results show changes in social interaction based on dual color optogenetic stimulation, highlighting differences in social behavior between the experimental and control groups. Red light stimulation resulted in increased social interaction, while blue light stimulation led to a decrease, demonstrating bidirectional control of behavior. (I to K) Reproduced from Ref. [126], with permission from Springer Nature.

In recent years, extensive research has been conducted on wireless optogenetic systems to enable unrestricted behavioral experiments and facilitate long-term neuromodulation studies. By eliminating the physical tethering constraints associated with traditional setups, these systems have aimed to achieve completely naturalistic behavior in experimental animals, opening new possibilities for understanding neural circuits and developing therapeutic interventions. Various wireless power transfer methodologies have been developed, including the use of batteries, magnetic coils, and RF antennas to power these wireless devices [52,83,84,141,[168], [169], [170]]. In line with these advancements, Gutruf et al. developed a battery-free wireless power transfer system that utilizes magnetic resonant coupling at 13.56 MHz to power a fully implantable, battery-free optoelectronic device (Fig. 3A) [87]. This system enables angle- and position-independent wireless power transfer, allowing for precise control of light stimulation intensity and spatial distribution. The absence of a battery in this design significantly minimizes the weight and volume of the device, offering a distinct advantage over head-mounted systems, which often incorporate batteries that contribute to increased bulk and potential immune response risks. However, the system's operation is limited to environments with compatible magnetic resonant setups, which can constrain its applicability in space. To address these spatial restrictions, Park et al. combined a solar power system for wireless communication, developing a device that eliminates the need for battery replacement, thereby enabling chronic and semi-permanent use (Fig. 3B) [140]. This system uses highly efficient InGaP/GaAs tandem solar cells (∼18 % efficiency) integrated on a flexible polyimide substrate, enabling it to harvest light energy for recharging a battery without requiring replacement. By leveraging solar energy, the device achieves chronic operation, making it suitable for semi-permanent use in light-rich environments. While its space-free operational design highlights its potential for long-term neuromodulation applications, it is essential to consider the immune response risks associated with the head-mounted system design. Unlike the previously discussed battery-free fully implantable and solar-powered systems, Kim et al. proposed a different approach using inductive power transfer to recharge a battery embedded in a soft, fully implantable optoelectronic device (Fig. 3C) [139]. This system combines the advantages of both battery-powered and battery-free designs, providing reliable wireless recharging while maintaining operational independence. The device can be charged wirelessly using a coil antenna and then operate without requiring continuous power transfer. Additionally, its programmability and ability to operate independently of specific environments enhance its utility for chronic in vivo studies. This system represents a distinct advancement, as it not only integrates the strengths of the two previous systems but also highlights the advantages of fully implantable designs for seamless and long-term usage in behavioral research.

Neural probe design allows for the precise insertion of a device into specific brain regions, enabling the measurement of signals or the delivery of stimulation to targeted areas [161]. A key advantage of integrating microfluidic channels into these neural probes is the ability to deliver drugs directly to the required site, minimizing off-target effects and reducing potential side effects [171]. When combined with optogenetics, this integration leads to the development of optofluidic systems, which offer several advantages. These systems allow for simultaneous optical and chemical modulation, providing a more versatile approach to manipulating neural circuits. They enable precise control over neural activity by delivering light to modulate specific neural populations while concurrently administering pharmacological agents to modulate broader neural responses. This dual-modality approach facilitates the study of complex neural dynamics, enhances flexibility in behavioral research [80,84,172]. Jeong et al. introduced a wireless optofluidic device that integrates both optogenetic stimulation and drug delivery via microfluidic channels into a single platform (Fig. 3D) [173]. The device uses polydimethylsiloxane (PDMS)-based soft, flexible microfluidic channels with a modulus of ∼1 MPa and bending stiffness of 13–18 N/m, making it mechanically compliant and suitable for minimally invasive use in neural tissue (Fig. 3E). The μ-LEDs are integrated onto a PET-based substrate, allowing for spatially and temporally precise light delivery to the outlet of the microfluidic channels. The embedded drugs are released through a thermal expansion mechanism triggered by Joule heating within connected reservoirs. The drug release performance was validated using an agarose gel brain tissue mimic and colored dyes, demonstrating the system's ability to achieve controlled drug release (Fig. 3F). The optofluidic capabilities of the device were tested in a place preference experiment (Fig. 3G). In the TH-Cre VTA-ChR2 mouse model, light stimulation of the VTA region typically induces a preference behavior. To evaluate the combined effect of optofluidic system, the dopamine antagonist SCH23390 was delivered to the target site, which resulted in the suppression of the light-induced preference behavior. The results confirmed that while preference was observed with only μ-LED activation, it was effectively blocked with simultaneous SCH drug delivery (Fig. 3H). This combined approach enables simultaneous pharmacological and optical manipulation within neural circuits, providing a deeper understanding of the complex interactions within these circuits. Additionally, the wireless programmable optofluidic system represents a significant advancement by enabling precise, untethered control of neural activity and drug delivery in freely moving animals.

Traditional neural probes integrated with μ-LED have been limited to using a single wavelength of light for neuromodulation. This restriction means that only one function—either inhibition or activation—can be controlled at a time, leading to limitations in behavioral experiments where only a single activity can be modulated. Such constraints have posed challenges in studying complex behavioral patterns and social interactions [[174], [175], [176]]. In contrast, Li et al. introduced a device that combines red and blue μ-LEDs into a vertical assembly, enabling simultaneous activation and inhibition within the same brain region (Fig. 3I) [126]. This device is fabricated on a flexible polyimide substrate, with the red μ-LED, optical filter, and blue μ-LED sequentially transfer-printed. SU-8 is used between each layer to ensure electrical insulation while maintaining optical transparency. The entire device is further encapsulated with PDMS and parylene, enhancing its waterproofing and flexibility, while providing sufficient bending stiffness to allow smooth insertion into brain tissue. The LEDs used in this device are 125 × 180 μm^2^ in size, specifically designed to target the VTA of mice. The use of an optical filter in the device, structured on multilayered titanium dioxide and silicon dioxide, allows for the selective transmission and reflection of specific wavelengths, optimizing the efficiency of optical stimulation from the red and blue μ-LEDs. The vertically assembled device using these optical filters for integrating both blue and red μ-LEDs has the advantage of requiring smaller space and providing an optimal profile for dual-color stimulation compared to conventional devices that are aligned in a lateral configuration [177]. Additionally, the copper coating on the polyimide substrate serves as an effective heat sink, reducing heat generation during prolonged or high-power light stimulation, thus preventing damage to brain tissue. The dual-color functionality of this device permits neuronal activation using the red μ-LED and inhibition using the blue μ-LED, enabling real-time social activity experiments that were previously impossible with single-wavelength stimulation. The device was implanted into mice with co-expression of ChrimsonR, which responds to red light, and stGtACR2, which responds to blue light, in the VTA. Observations of social activities under different stimulations revealed that red LED stimulation promoted social interaction, while blue LED stimulation suppressed it (Fig. 3J and K). The bidirectional optogenetic modulation capabilities of the device introduced in this study overcome the limitations of traditional unidirectional stimulation methods, demonstrating potential for expansion into various in vivo experiments, including complex social behavior studies [178].

##### 3.1.1.2 Surface mount device

In addition to LED probes designed for deep brain insertion, there are also LED devices engineered to attach to the surface of the brain [60,119,150,[179], [180], [181], [182]]. These surface-mounted LED devices, often used for non-invasive or minimally invasive optogenetic stimulation, offer unique advantages. Unlike penetrating probes, these devices are placed directly on the cortical surface conformally, avoiding the risk of damaging deep brain structures [183]. This approach is particularly effective for stimulating large cortical areas, making it ideal for applications such as studying neural networks involved in sensory processing, motor control, and cognitive functions [179,180,184].

One of the critical design considerations for surface-mounted LED devices is flexibility and biocompatibility. These devices must conform to the brain's complex, dynamic surface without causing irritation or damage. In addition, strong adhesion between the device and the brain surface is crucial for preventing slippage and ensuring precise light delivery to targeted regions [185]. Thinner films allow for better conformity to the brain's irregular surface, improving contact, while materials with appropriate mechanical properties, such as low stiffness and high flexibility, ensure that the device can adhere securely without causing damage or discomfort to the surrounding tissue [186,187].

In brain surface optogenetics, wide-area coverage is particularly important due to the large-scale and distributed nature of cortical functions such as sensory processing, motor control, and cognition. Stimulating broad cortical areas enables activation of entire neural circuits, essential for understanding complex behaviors and cortical coordination [60,180,182]. Additionally, enhanced spatial resolution of LEDs allows for more precise control of specific neuronal populations, minimizing off-target effects and enabling more detailed investigation of neural pathways [188]. For these reasons, extensive research has focused on improving both wide-area coverage and spatial resolution to advance cortical optogenetic studies.

**Fig. 4:**
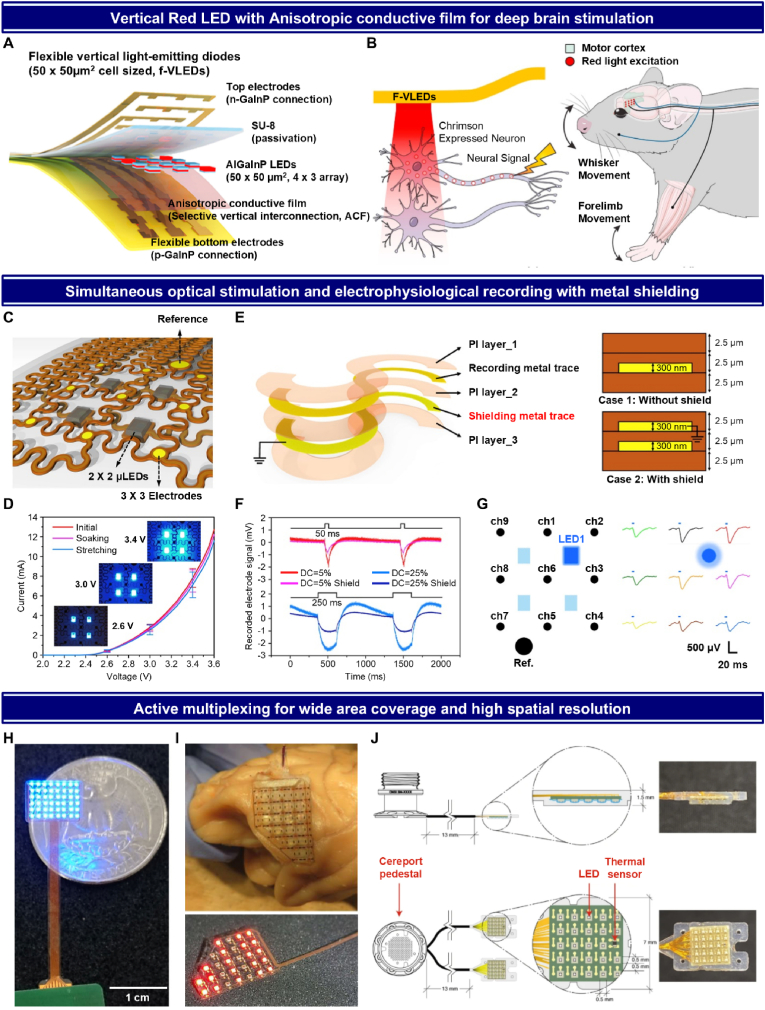
Various advanced optogenetic devices on surface region of the brain. (A) Layered structure of flexible vertical LEDs (f-VLEDs) with anisotropic conductive film (ACF) for enhanced current spreading and heat dissipation, enabling efficient stimulation of deep cortical layers while conforming to the brain surface. (B) In vivo application of the red-light-emitting AlGaInP LED array, demonstrating successful induction of whisker and forelimb movements. The high-power red-light LEDs (638 nm) in combination with Chrimson, a red-shifted ChR, allowed deeper cortical layer stimulation, enabling precise neuromodulation of motor responses from the brain surface. (A to B) Reprinted from Ref. [179], Copyright (2018), with permission from Elsevier. (C) Schematic layout of the stretchable opto-electric neural interface (SOENI), integrating μ-LEDs and recording microelectrodes on a flexible, serpentine-shaped structure, designed for optimal conformation to the brain surface during optical stimulation and ECoG recording. (D) Current-voltage (I–V) characteristics of the SOENI during initial, soaking, and stretching conditions. The graph shows stable performance across multiple conditions, including soaking at 75 °C for 7 days and repeated 10 % mechanical stretching for 500 cycles. (E) Cross-sectional illustration (left) of the SOENI layers, including recording and shielding metal traces, and a comparison (right) between cases with and without the metal shielding layer. (F) In vitro comparison of noise artifacts caused by active μ-LEDs at different duty cycles (5 % and 25 %) and frequencies (1 Hz–20 Hz). The metal shielding layer, grounded for optimal EMI reduction, decreased the peak-to-peak voltage of noise artifacts by over 50 %, improving the signal-to-noise ratio and ensuring clearer neural recordings during optogenetic stimulation. (G) In vivo optical stimulation showing evoked negative potentials from multiple channels. Higher potentials were recorded near the active μ-LED (LED1), demonstrating the SOENI's high spatial resolution for precise cortical stimulation. (C to G) Reprinted from Ref. [119], Copyright (2020), with permission from Elsevier. (H) Planar μ-LED array device featuring a 6 × 8 array of 48 blue LEDs, designed for wide-area cortical stimulation in macaque brains. The flexible polyimide substrate includes holes for microdialysis probes, enabling simultaneous photo-stimulation and neurotransmitter measurement. © [2021] IEEE. Reprinted, with permission, from Ref. [180]. (I) Optical images of flexible red-light LED array designed for wide-area coverage and deep brain stimulation in primates, enabling precise targeting of regions like the VTA with enhanced tissue penetration compared to blue-light systems. From Ref. [182]. Reprinted with permission from AAAS. (J) schematic illustrating a chronically implantable 2D matrix of LEDs, combined with a thermal sensor for simultaneous temperature monitoring in non-human primates. Reproduced from Ref. [60], with permission from Springer Nature.

Surface-mounted film-type devices offer a less invasive alternative to penetrating probes for optogenetic studies, yet they often face limitations when using conventional ChR paired with blue-light LEDs. These blue-light systems primarily stimulate only the superficial layers of the cortex [99], restricting their effectiveness in influencing deeper regions. The cortex is a highly structured and layered region of the brain, comprising multiple layers and distinct functional regions [189,190]. Stimulating multiple layers and regions simultaneously can provide comprehensive insights into complex neural circuits [191,192]. Lee et al. addressed this limitation by developing a red-light-emitting AlGaInP LED device, coupled with the red-shifted ChR, Chrimson [179]. This combination enables the simultaneous stimulation of multiple cortical layers and regions, offering a broader and deeper reach within the cortex. The AlGaInP LEDs (50 × 50 μm^2^, 4 × 3 array) emit light at a wavelength of 638 nm, achieving a power density of over 25 mW/mm^2^, sufficient to penetrate and stimulate deeper cortical layers compared to traditional blue-light systems (Fig. 4A) [105]. Additionally, the device employs an anisotropic conductive film (ACF) to form vertical LED interconnections, which improves current spreading and heat dissipation, key advantages of the vertical LED structure [193]. Despite this configuration, the device maintains a thin profile of approximately 35 μm, ensuring excellent flexibility and allowing it to fit smoothly inside the skull while conforming closely to the brain's surface [194]. Fig. 4B shows the in vivo application of this device. When placed on the cortical surface, the red-light LEDs successfully induced whisker and forelimb movements in mice, demonstrating the device's ability to effectively target and modulate specific neuronal populations. This approach highlights the advantages of using red-light optogenetic tools for more comprehensive and less invasive neuromodulation.

When optogenetics is combined with simultaneous electrophysiological (EP) recording, it allows researchers to precisely control and observe neural activity, providing deeper insights into brain functions and neural circuits [195,196]. To harness these advantages, Ji et al. developed a stretchable device capable of performing both electrocorticogram (ECoG) recording and optical stimulation in the cortex of mice (Fig. 4C) [119]. This device integrates four μ-LEDs for optogenetic stimulation and nine electrodes for neural signal recording, designed with a serpentine-shaped structure that enhances the device's flexibility and stretchability, allowing it to conform to the brain's surface. It is constructed using ultra-soft materials like Dragonskin silicone elastomer, which further improves its mechanical properties, ensuring secure adhesion to the brain without causing irritation or damage. The electrical robustness of this design is demonstrated in Fig. 4D, where the LED functionality remains stable even after soaking and mechanical stretching, confirming its durability for long-term implantation.

When optogenetic stimulation and EP recording are performed simultaneously, electromagnetic interference (EMI) from the LEDs can distort the recorded signals [197]. To address this, Ji et al. introduced a metal shielding layer between the recording electrodes and the LEDs (Fig. 4E) [119]. This shielding significantly reduces EMI, which becomes especially important during higher duty cycles of LED stimulation. The effectiveness of the shielding is demonstrated in Fig. 4F, where the recorded signals show much lower noise levels when the shielding layer is used, particularly at 25 % duty cycle, confirming that the metal shield effectively mitigates EMI during optogenetic stimulation. The in vivo experiment validated the device's robustness, where optical stimulation from the μ-LEDs was applied to the cortex of awake mouse. The experiment successfully recorded high-quality neural signals with excellent spatial resolution, demonstrating the system's ability to stimulate and monitor neural activity simultaneously (Fig. 4G). This experiment confirms the viability of the device for advanced neuroscience research.

In contrast to deep brain optogenetics, which typically targets localized regions, surface-mounted optogenetic devices often cover broader areas of the brain, particularly in regions like the cerebral cortex. This is essential for studying large-scale neural networks involved in sensory processing, motor control, and cognition [60,180,182]. When transitioning from small animal models like rodents to larger primates such as macaque monkeys, and eventually humans, achieving wide-area coverage and enhanced spatial resolution becomes crucial [115]. In the context of larger brains, LED array devices must be designed to stimulate vast cortical surfaces with high precision.

Devices like the μ-LED array introduced by Ohta et al. are examples of design rules that achieve this balance (Fig. 4H) [180]. This array is flexible and biocompatible, capable of adhering to curved cortical surfaces. Its high-density LED layout enables stimulation of multiple points while providing wide coverage with precise control. Active multiplexing—independent control of numerous LEDs—allows researchers to toggle specific areas of the brain on and off, maximizing flexibility and spatial resolution. The multi-LED device in Sasaki et al. extends this approach by employing red-shifted light to reach deeper brain regions, such as the VTA (Fig. 4I) [182]. This is especially important in primates and humans, where deeper structures must be stimulated to explore motivation and reward circuits. Red-light LEDs penetrate deeper tissue, offering a solution to the limitations of blue-light systems, which primarily affect superficial layers. Both Ohta's and Sasaki's devices employ active multiplexing to stimulate different parts of the brain independently, enhancing both precision and area coverage. Rajalingham et al. further demonstrated this with the Opto-Array, designed for chronic use in primates (Fig. 4J) [60]. The array stimulates large areas of the visual cortex and includes thermal sensors to ensure safe, long-term use. Its capacity for wide-area stimulation, combined with consistent performance across experimental sessions, highlights its utility for extended research.

#### 3.1.2 Spinal cord

The spinal cord is fundamental in transmitting information between the brain and the rest of the body, containing a variety of neuronal and non-neuronal cells such as motor neurons, sensory neurons, interneurons, visceral afferent/efferent fibers, and glia [198]. Spinal cord neuromodulation is used to manage chronic neuropathic pain and improve motor functions by modulating the EP signals in specific regions of the spinal cord [[199], [200], [201]].

The spinal cord is enclosed by a structure composed of the dura mater, adipose tissue, and bone [202]. For spinal cord neuromodulation, devices are typically inserted into the epidural space, located below the dura mater that surrounds the spinal tissue, allowing precise modulation at the targeted region [203,204]. As such, the limited anatomical space of the epidural region must be considered when designing the dimension of spinal implant devices [205]. Another critical point in device design is minimizing biomechanical mismatch between the implants and neural tissue [206]. Neural tissue exhibits extremely soft and has a low modulus, so devices must be engineered to mimic the mechanical properties of the implantation site to minimize immune responses and ensure long-term stability [202]. By aligning the shape and mechanical behavior of the implant with the spinal tissue, static and dynamic mechanical properties can be matched, reducing mechanical mismatch and enhancing the device's longevity and effectiveness [207,208].

The spinal cord structure includes the dorsal horn and ventral horn, each with distinct functions related to sensory and motor activities [209]. The dorsal horn primarily processes sensory information and is significantly involved in pain perception [210]. In contrast, the ventral horn contains motor circuit neurons that regulate movement behaviors [211]. Additionally, the most abundant neurons in the dorsal horn are interneurons, which influence both sensory and motor functions by balancing excitation and inhibition within the spinal circuit [212]. Since the modulation functions vary according to the depth of the spinal tissue, and the implant area of the device is the epidural space of the spinal cord, it is difficult to deliver sufficient optical power to the ventral horn region of the spinal cord. Therefore, increasing the number of LEDs or using red-wavelength light, which penetrates deeper regions, can facilitate better modulation in deeper region of the spinal tissue [53]. However, careful control of temperature changes due to the increased number of LEDs is necessary to prevent issues related to overheating [213].

**Fig. 5:**
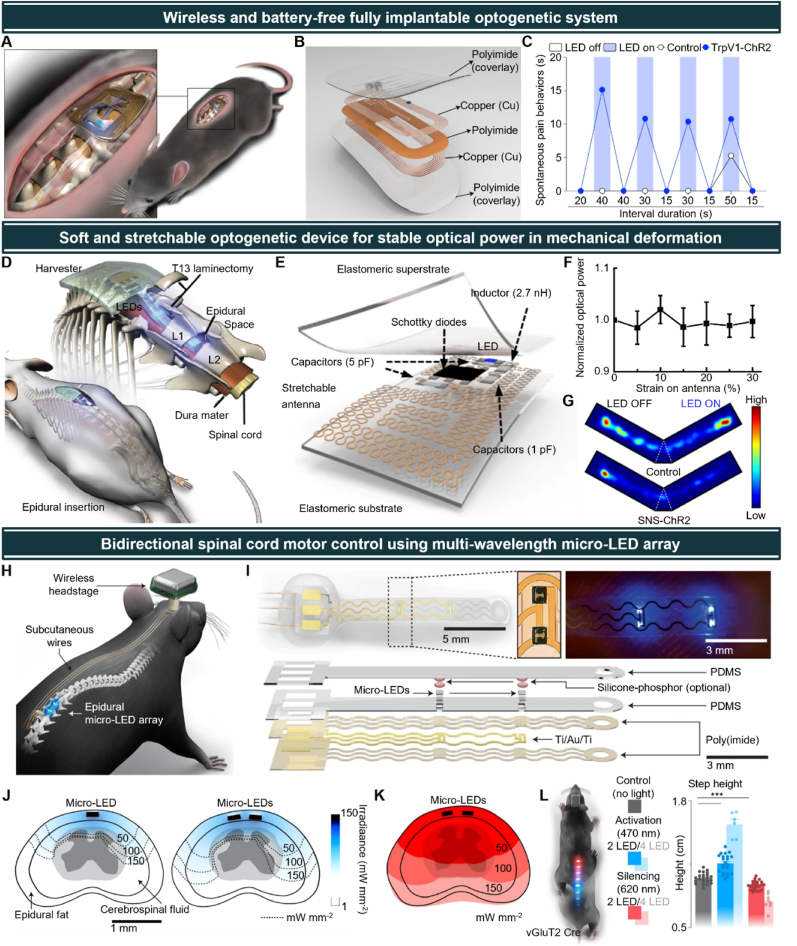
Various advanced optogenetic devices on spinal cord. (A) Illustration of the inserted location of the wireless and battery-free fully implantable optogenetic spinal neural implant. Positioned in the epidural space, the device enables precise spinal neuron control using RF antenna-based power harvesting for long-term operation without external wires or batteries. (B) Schematic illustration of the spinal implant layer structure, including an RF coil for power harvesting. The implant efficiently captures wireless energy at 13.56 MHz to power its optogenetic components, enabling stable operation without a battery. (C) Pain-like behavior in TrpV1-ChR2 mice and control mice during optical stimulation. Blue light indicates the LED on state, activating TrpV1-ChR2 channels and inducing nociceptive responses in the experimental group, while control mice show no significant behavioral changes. (A to C) Reproduced from Ref. [216], with permission from Springer Nature. (D) Illustration of the anatomy and location of the soft and stretchable epidural optogenetic device. The device is implanted in the epidural space along the spinal cord, designed to conform to the spinal curvature while maintaining stable optical stimulation. (E) Schematic illustration of the stretchable energy harvester component with integrated IC and LED. The stretchable design allows the system to maintain electrical performance under mechanical strain, ensuring stable power delivery even during spinal cord movements. (F) Normalized optical power in response to strain on the stretchable antenna, demonstrating stable optical output up to 30 % strain, even in a worst-case scenario. This highlights the device's robustness, maintaining consistent performance under significant mechanical stress. (G) Behavioral response in SNS-ChR2 mice during Y-arm maze test under LED ON and OFF conditions. In the control group, no preference is observed. However, SNS-ChR2 mice exhibit a preference for the LED OFF location due to nocifensive behavior triggered by LED ON stimulation. (D to G) Reproduced from Ref. [120], with permission from Springer Nature. (H) Illustration of the epidural μ-LED device for spinal cord motor control. The epidural array is implanted along the spinal cord and connected via subcutaneous wires to a wireless, head-mounted head stage. (I) Schematic illustration of the soft optoelectronic implant layer structure, including a μ-LED array. Designed for chronic implantation, the flexible layers conform to the tissue and maintain stable optogenetic stimulation over long periods, minimizing tissue damage and ensuring reliable, long-term neuromodulation. (J) Finite element model comparing the blue light penetration depth of a single μ-LED and an array of μ-LEDs within the spinal cord. The model demonstrates how the irradiance differs between individual and multiple μ-LEDs, providing insights into optimizing light delivery for effective neuromodulation. (K) Simulation of the red-shifted light penetration depth within the spinal cord. The longer wavelength of red light allows for deeper tissue penetration, effectively reaching the ventral horn. This enhanced irradiance ensures more efficient neuromodulation in deeper regions. (L) Mice expressing ChR2 and Jaws in vGluT2ON receive blue and red optical stimulation while stepping on a treadmill. Blue light increases step height by activating excitatory neuromodulation, while red light reduces step height through inhibitory neuromodulation, demonstrating bidirectional control of motor function. (H to L) Reproduced from Ref. [53], with permission from Springer Nature.

Similar to the brain, the application of wireless systems to the spinal cord offers significant advantages. Traditional spinal cord neuromodulation using wired optical power sources requires an external tethered system, posing a risk of infection at the implant site and hindering natural movement [214]. Additionally, head-mounted systems further amplify these risks due to their design, which involves externalized devices that remain outside the body. This can lead to grooming issues, increased susceptibility to infections, and limitations in experimental paradigms, often restricting studies to single subjects. In contrast, fully implantable wireless systems reduce these risks, promoting safer, more flexible neuromodulation by allowing natural behaviors and eliminating the need for external connections [215]. Grajales-Reyes et al. introduced fully implantable wireless optogenetic device designed to minimize movement restrictions and risk of infection (Fig. 5A) [216]. The entire device is designed with a probe shape with an LED, housed within a rectangular-shaped coil for power harvesting. The device is designed in a probe form, with the LED integrated into the probe to be inserted into the epidural space, accounting for its spatial constraints to facilitate effective neuromodulation. The rest of the device's circuit is positioned above the spinal cord, outside the epidural space, to optimize functionality while minimizing intrusion. The device layout is constructed with a flexible polyimide-based substrate, coated with PDMS to protect external electronic components, and fully encapsulated with parylene for durability (Fig. 5B). Notably, the mechanical flexibility and thin form factor allow the device to maintain close contact with the spinal cord, improving the precision of optical activation. Besides, the device utilizes near-field communication (NFC) for wireless power transfer, harvesting energy from magnetic fields generated by an external antenna through a rectangular coil. The received AC signal is converted into a DC signal via a rectifier circuit, powering the μ-LED. To validate the robustness of this system, the device was implanted beneath the epidural space of the spinal cord in TRPV1-ChR2 mice for optogenetic studies. LED activation evoked significant pain behaviors in ChR2-expressing mice, whereas control mice did not exhibit any pain responses during the same stimulation (Fig. 5C).

Compared to brain neuromodulation, spinal cord neuromodulation involves greater mechanical deformation of the device due to the movement of the vertebrae. The spine consists of multiple vertebrae that move individually within a dynamic environment to accommodate various body movements [217,218]. Therefore, the mechanical stresses applied to the device during spinal cord neuromodulation must be thoroughly considered in the design process [219]. As a result, the efficiency of wireless power transfer fluctuates, making it difficult to maintain consistent optical power output from the LED. This can lead to unintended experimental outcomes due to changes in the LED optical power or prevent accurate optical stimulation, ultimately making it challenging to conduct precise behavioral experiments. Park et al. introduced the device that is based on a low-modulus silicone elastomer platform and utilizes a stretchable antenna for wireless power transfer, allowing stable LED operation even under mechanical deformation caused by the movement of the mouse (Fig. 5D) [120]. The device incorporates serpentine-shaped electrical interconnects and is encapsulated with polyimide and low-modulus silicone elastomer. These design elements emphasize the importance of low-modulus materials and the stretchable antenna, which are critical for achieving reliable wireless operation and successful long-term implantation (Fig. 5E). The miniaturization of the device relies on a stretchable antenna that captures RF power via capacitive coupling between adjacent patterns, making it highly suitable for implantation in challenging areas. The soft, system-level mechanics resulting from this design, with an effective modulus of approximately 1.7 MPa, allow the device to conform to anatomical shapes and natural body movements. This low-modulus platform is key to enabling stable, fully implantable device performance over extended periods. Even when the device is twisted, the LED continues to operate normally, demonstrating the robustness of the design. These mechanical optimizations ensured reliable activation of the device even after six months of implantation. The design also accounts for strain induced by movements, allowing the device to maintain functionality even under extreme strain scenarios (up to 30 %). Furthermore, even with up to 30 % strain applied to the antenna, the normalized optical power shows minimal variation, indicating stable and reliable device functionality under mechanical strains (Fig. 5F). To evaluate the performance of the device, it was implanted in the spinal cord epidural space of SNS-ChR2 mice to assess nocifensive behavior triggered by LED stimulation. In the Y-Arm maze test for behavioral aversion, it was observed that while the control unit showed no preference between LED on/off regions, SNS-ChR2 mice exhibited significant aversion to the LED ON region (Fig. 5G). These experimental results highlight the device's unique advantage of providing precise neural stimulation with stable optical power and functionality, even under mechanical deformation and long-term implantation, making it a robust and reliable solution for advancing neuromodulation research and clinical applications.

In spinal cord neuromodulation, effective motor control requires light to reach the deeper region of the spinal tissue, specifically the ventral horn, to stimulate motor neurons and interneurons. However, with blue light, tissue penetration depth is typically very shallow, making it difficult to deliver sufficient optical power to deeper regions [220]. To solve this problem, Kathe et al. presented a wireless optogenetic system controlling mouse locomotion by using blue LEDs for activation and red LEDs for inhibition (Fig. 5H) [53]. The device was designed using a combination of thin-film microtechnology, elasticity engineering, μ-LEDs, and dura-like silicone, resulting in a chronically implantable epidural system. The system utilizes an elastic serpentine structure made of titanium/gold/titanium (Ti/Au/Ti) metal embedded in a polyimide-based substrate. The μ-LEDs are coated with a silicone-phosphor composite, and the entire device is encapsulated in a silicone membrane, achieving mechanical compliance similar to that of the natural dura mater (Fig. 5I). This design allows the device to remain stable under strains up to 15 %, which is greater than the typical displacements experienced by the spinal cord. Reliability tests conducted under accelerated aging conditions confirmed that the device maintains stable electrical properties for at least 30 days, indicating that it can function in vivo for several weeks. To validate whether the deeper regions of ventral region in the spinal cord can be adequately stimulated, finite element modeling was used to assess light penetration within the spinal cord. The results showed that while a single blue μ-LED was insufficient to effectively stimulate spinal interneurons, using two blue μ-LEDs successfully delivered enough optogenetic stimulation to both afferent fibers and spinal interneurons, enabling motor control (Fig. 5J). Also, simulations with red LEDs confirmed that red-shifted light can sufficiently deliver the optical power to target motoneurons for motor control (Fig. 5K). To validate the two-color optogenetic activation and inhibition, the device implanted on the mice that expressing both ChR2 (470 nm) and Jaws (600 nm) in vGlu2ON neurons (Fig. 5L). The μ-LED array was strategically positioned along distinct rostrocaudal locations. Blue light activation elicited strong muscle responses, leading to an increase in step height. Conversely, inhibition with red-shifted light reduced the vigor of locomotor movements, resulting in a decrease in step height. These results demonstrate the system's ability to achieve precise and comprehensive entire dorsoventral spinal modulation, enabling targeted motor control through both activation and inhibition with dual wavelength optogenetic control in freely moving mice.

### 3.2 Other organs and peripheral nervous system (PNS)

#### 3.2.1 Organs (heart, bladder, stomach)

Organs carry out specific functions essential for survival, such as blood circulation, digestion and excretion. Organs in the human body include the heart, lungs, stomach, and bladder. These organs are part of larger systems, such as the cardiovascular, respiratory, digestive, and urinary systems. The stimulation of organs is primarily achieved through two methods: directly targeting the cells present on the organ's surface or stimulating the autonomic nerves connected to the organ. Directly targeting composites of organs requires consideration of unique characteristics of the organs, such as their morphology, size, or light permeability. Especially, since autonomic function of organs are usually driven by movement of muscles, mechanical properties including stretchability must be significantly considered. These factors must be accounted for in the design of appropriate optogenetic devices.

Optogenetic interventions aimed at modulating cardiac functionality predominantly seek to address arrhythmia [[221], [222], [223]], a disorder characterized by abnormal heart rhythm. Mouse's heart typically has a diameter of approximately 7 mm [224], with its wall composed of rod-shaped cardiomyocytes ranging from 100 to 150 μm in length and 10–25 μm in width. Considering that neurons have a radius of about 10 μm, relatively large size of cardiomyocytes may provide an advantage in terms of targeting individual cells, minimizing interference of LED light on peripheral cells. Another distinguishing feature of cardiac muscle cells lies in their mode of intercellular signaling. While neurons mostly rely on synapses to transmit signals via neurotransmitter release across spatial gaps, cardiomyocytes establish direct cytoplasmic connections through gap junctions. This direct coupling allows for the rapid propagation of electrical signals between cells, facilitating the synchronized contraction of cardiomyocytes. Such coordination is essential for the efficient pumping of blood by the heart, ensuring that cardiac muscle cells contract in unison. Thus, as using a single LED to stimulate the heart delivers signals to only a limited area, it may potentially disrupt the synchronized activity of cardiomyocytes. Therefore, to achieve synchronized activation over a larger portion of the heart, the LED array should cover a wide region, enabling simultaneous stimulation of multiple cardiac regions.

The stretching movement of heart results in volume change between the systole and diastole periods, approximately 32 % in rats [225]. This significant stretchability of the heart can limit the deployment of materials with low yield strength, as devices made of these materials may dislocate from the heart, impeding conformal operation. Therefore, when designing a device to be attached to the surface of the heart, materials that are sufficiently stretchable to endure significant strain should be used. Alternatively, stretchability can be achieved by employing structural features on non-stretchable materials, with serpentine design being a prime example [170,226].

**Fig. 6:**
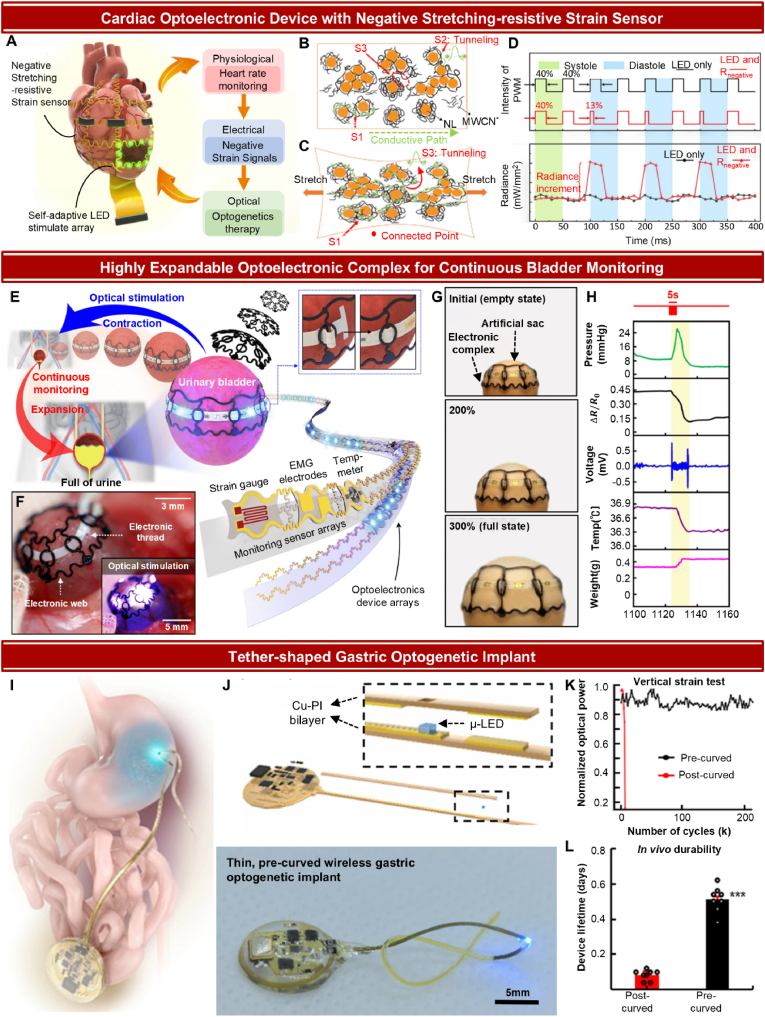
Various advanced optogenetic devices on autonomic nervous Systems. (A) Schematic diagram of closed-loop cardiac optogenetic modulation system. The device is composed of a negative stretching-resistive strain sensor and LED array. Negative strain sensor acquires strain map during systole and diastole period of heart, monitoring heart rate of patient. When abnormal heartbeat frequency has been reported, the system subsequently delivers electrical signals to LED array, turning on LED for cardiac pace making using optogenetics. (B and C) Principles of negative stretching-resistive effect of CNT-NL.: (B) unstretched (top), (C) stretched horizontally (bottom). Three types of conductive situations at unstretched state: conductive by CNT connections (S1), minor gap for electron tunneling (S2), nonconductive path due to large gap (S3). When stretched, S1 and S2 are compressed strongly enough to form conductive path, and the gap on S3 becomes minor, inducing tunneling effect. (D) Duty cycle (top) and radiance (bottom) of current-type μ-LED. Duty cycle was set to be 40 % in systole period and adjusted to 13 % in diastole period, rather than retaining high duty cycle for whole operation time. μ-LED with negative stretching-resistive strain sensor (R_negative_) exhibited increment in radiance from 74.94 to 89.41 mW/mm^2^ on diastole period, while no significant change shown without the strain sensor (LED only). (A to D) From Ref. [227]. Reprinted with permission from AAAS. (E) Illustration of optoelectronic complex. E-thread consists of LED array, strain gauge sensor, EMG electrodes and temp-meter, enabling multifunctional signal acquisition and optical stimulation simultaneously. Inset describes how E-web sustains conformal contact of E-thread with bladder. The multifunctional system continuously monitors physiological signals of bladder during expansion and urination. (F) Optoelectronic complex fixed on mouse bladder. Inset: operation of μ-LED for optical stimulation. (G) Optoelectronic complex on artificial sac. The complex successfully secured conformal contact with the 300 % enlarged sac. (H) Multimodal measurement of physiological signals on bladder. 5s Optical stimulation (red) induced variations in intravesical pressure (green), strain (black), EMG (blue), temperature (pruple), and micturition weight (magenta). (E to H) From Ref. [230]. Reprinted with permission from AAAS. (I) Schematic of tether-shaped optogenetic device stimulating vagus nerve connected to stomach. (J) Structure of the device (Top), Inset shows how μ-LED is sandwiched between two Cu-PI layers; Tethers are pre-curved according to its application region before encapsulation (bottom). (K) Vertical strain cycling test of pre-curved, post-curved device. The pre-curved device retained its optical power up to 200k cycles, while post-curved device lost is function at 6k cycles. (L) In vivo durability test showing pre-curved tether has advantage regarding on lifetime over post-curved tether. (I to L) Reproduced from Ref. [234], with permission from Springer Nature.

Optogenetic applications in organs, including cardiac systems, frequently employ strain gauge sensors in conjunction with μ-LEDs to measure the degree of organ stretching in real time [153,227]. The strain mapping serves as a critical parameter for detecting abnormal pumping action. By developing a closed-loop system that determines μ-LED illumination based on the strain mapping, it is possible to deliver targeted treatment at the optimal time, thereby enhancing the effectiveness of the intervention and improving overall device performance. Moreover, the gauge factor of the materials used in the strain sensor must be carefully considered. A positive gauge factor can result in a positive stretching-resistive effect, increasing the resistance of the μ-LED interconnects when stretched, which may diminish light intensity. In contrast, using materials with a negative stretching-resistive effect can retain stable light intensity of μ-LEDs above threshold under stretching with the same power input. This characteristic is particularly crucial for cardiac pace-making applications, where consistent levels of stimulation are required for patient's safety. Hong et al. introduced a bidirectional device that integrates a negative gauge factor strain sensor with μ-LEDs (Fig. 6A) [227]. Materials exhibiting a negative stretching effect were achieved by using natural latex (NL) combined with carbon nanotubes (CNTs). The conductive CNTs form a core-shell structure, encapsulating NL to create individual particles. In the unstretched state, sparse distribution of particles hinders CNTs establishing a complete conductive path (Fig. 6B). However, when the device is stretched, extrusion occurs perpendicular to the stretching direction, decreasing the inter-particle distance, allowing CNTs to form connected conductive paths efficiently (Fig. 6C). Consequently, the negative gauge factor in the strain sensor led to increased radiance in the stretched state compared to the original state.

The device's application in regulating cardiac function was validated through in vivo experiments performed on mice injected with AAV2/9-CAG-ArchT-GFP. The strain sensor monitored the heart's cycle length, which refers to the time interval between pumping actions, and automatically activated the μ-LED when the cycle length shortened, which indicates the onset of ventricular tachycardia (VT). The device utilized current type μ-LEDs, and duty cycle was adjusted during diastole period, rather than sustaining uniform duty cycle over operating time. This led to an increment of radiance during diastole period when negative stretching-resistive strain sensor was applied, while no radiance increment was shown without the strain sensor (Fig. 6D). The μ-LED device operated with low power consumption and effectively addressed VT by silencing muscle activation via ArchT, resulting in an elongation of the cycle length by up to 58 %.

Similar to heart, bladder function mainly relies on contracting and stretching movements of muscles. Bladder serves as a dynamic reservoir for urine storage and controlled excretion. This muscular sac accommodates increasing urine volumes through its remarkable distensibility. Optogenetic applications targeting the bladder focus primarily on the control of micturition by stimulating detrusor muscle, which is responsible for bladder contraction [228]. The detrusor muscle is located just beneath the outermost layers of the bladder wall, which are only 20–50 μm thick in mouse [229] and allow for the penetration of light wavelengths commonly used in optogenetics, ensuring effective regulation of bladder movements through surface-mounted device.

The bladder experiences extensive volume increase from empty state when fully distended. In terms of deformation, the bladder undergoes greater volume changes than the heart [101,226]. However, unlike the heart, which has rapid contraction and relaxation cycles, the bladder has longer intervals between voiding cycles. This indicates that the bladder-targeting devices are subjected to fewer strain cycles, resulting in less frequent mechanical stress compared to cardiac devices.

Jang et al. designed a multimodal device capable of simultaneous stimulation and comprehensive monitoring, taking into account the extensive stretchability of the bladder [230]. The device is primarily composed of two key components: electronic thread (E-thread) and electronic web (E-web) (Fig. 6E and F). The E-thread is equipped with μ-LEDs, electromyography (EMG) sensors, a strain gauge sensor, and a temperature sensor, enabling optogenetic stimulation while simultaneously monitoring multiple signals from the bladder. Although the interconnects were designed with a serpentine structure to enhance stretchability, the significant bladder's expansion during filling can cause detachment issues. To address this challenge, the E-web, made of a silicone elastomer, features a soft, expandable structure that seamlessly integrates the E-thread with the bladder, ensuring stable monitoring of physiological signals and consistent optoelectronic stimulation throughout the bladder's filling and voiding cycles (Fig. 6G).

The optical stimulation and monitoring capabilities of the device were validated using a Detrusor Underactivity (DUA) model. DUA mice are unable to sustain bladder contractions long enough to fully void due to neurogenic or myogenic disorders when no proper treatments are applied. Injection of ChR2 encoding adenovirus between detrusor muscle and adventitia secured uniform distribution of ChR2 on target region. Subsequently, the electronic complex conformally attached on bladder illuminated the target region, which induced normal muscle contractions, facilitating voiding activities. The multimodal sensor was able to monitor the changes in EMG, strain, and temperature that occurred during voiding activities, which provide more reliable bladder information and fundamental treatments incorporated with existing technologies (Fig. 6H).

Another optogenetic intervention strategy involves the targeted stimulation of autonomic nerves innervating specific organs. The ANS can be broadly classified into the sympathetic nervous system and the parasympathetic nervous system, each system interacting with the control centers such as brain and spinal cord. The sympathetic nervous system is primarily activated during stressful situations, while parasympathetic nervous system, on the other hand, is related to promotion of relaxation. The sympathetic nervous system signals pass through the spinal cord, and are delivered through series of nerves, traveling sequentially through preganglionic neurons, autonomic ganglia, and postganglionic neurons before connecting to respective target organs. Parasympathetic nervous system, on the other hand, is predominantly connected directly to the brain via cranial nerves. The one exception lies on sacral portion: lower parts of the body, including lower colon, rectum, bladder receive their signal via spinal cord [231].

Although nerves generally exhibit similar morphological characteristics, their diameters can vary significantly. For example, vagus nerve (Cranial Nerve X) typically has a diameter of 1.4 mm [232], whereas pre- and postganglionic neurons exhibit diameters of approximately 0.3–5 μm. While cuff electrode on the vagus nerve has been previously reported [233], the size disparity poses challenges for versatile application of cuff electrodes, as the smaller diameter of certain nerves makes secure attachment difficult. Therefore, when designing an optoelectronic device for thin nerves, mere positioning μ-LEDs in close proximity could be a viable alternative to using a cuff form.

Kim et al. developed a device featuring soft, low-modulus tethers embedded with μ-LEDs to directly stimulate the vagus nerve connected to the stomach (Fig. 6I) [234]. The μ-LEDs were first mounted on the tether's bottom layer, composed of a Copper-Polyimide (Cu-PI) bilayer, and then encapsulated by a top Cu-PI bilayer, sandwiching the μ-LED (Fig. 6J). Subsequently, tether was pre-curved before PDMS encapsulation process, which holds advantage against post-curved tether in terms of mechanical stability (Fig. 6K) and in vivo durability (Fig. 6L). One key feature of the device is that curving shape of tether can be specifically determined to match the anatomical structure of target region. This customization method offers the advantage of enabling nerve modulation in various locations with minimal modification to the device design, thereby enhancing its versatility for different applications.

Another key advantage of this μ-LED device is its wireless, fully implantable design. Utilizing an implanted wireless RF-powered energy harvesting system, the device operates without the need for external wiring. This wireless functionality effectively minimizes the potential risk of infections associated with external connections while also reducing movement restrictions for experimental subjects. Moreover, wireless operation has been successfully demonstrated in in vivo experiments, not only in the stomach but also in other organs such as the heart [170,226] and bladder [101], highlighting its versatility for various biological applications.

The μ-LED embedded tether was utilized on mouse to regulate its intake behavior. The μ-LED was specifically implanted on corpus region of stomach, where adenovirus has been introduced to express blue light-sensitive ChR2. Stimulation via μ-LED embedded tether significantly reduced cumulative intake compared to the control group, demonstrating that nerve stimulation connected to an organ can effectively induce behavioral changes.

#### 3.2.2 Somatic nervous system (SNS)

In contrast to the brain and spinal cord, which are protected and relatively stationary due to the cranium and vertebral column, the SNS presents unique challenges for implantable LED device design. The SNS is distributed across muscles, skin, and joints, making it subject to significant relative movement caused by bodily motions, external stimuli, and changes in body position. This dynamic environment creates distinct challenges for the stable and effective implantation of optogenetic devices targeting the SNS [235].

In addition to relative movement, the SNS is closely associated with muscles, meaning that when muscles contract and relax, nerves move as well [236]. This creates further complexity when trying to maintain the precise positioning of an LED device, as even small displacements can result in inconsistent light delivery, potentially reducing the effectiveness of neuromodulation. For this reason, a key design rule for LED devices targeting the SNS is ensuring stable and secure fixation at the implantation site [235,237].

Given these challenges, the design of LED devices for somatic neuromodulation must prioritize materials and anchoring mechanisms that can maintain the device's position even in the presence of muscle contractions and general body movement [142,238,239]. One possible approach is to use flexible, stretchable materials that can conform to the body's natural movements without causing discomfort or displacement [120]. These materials must also balance flexibility with structural integrity, ensuring that the device does not shift while still accommodating the movement of surrounding tissues.

Furthermore, the proximity of sensory nerves in the somatic system introduces another important design consideration. Unlike autonomic nerves, which control involuntary actions like heartbeats or digestion, the SNS governs voluntary movements and sensory processing. Therefore, any discomfort or irritation caused by an implanted device is more likely to be noticed by the user [240]. This heightened sensitivity makes it crucial to minimize any mechanical irritation or discomfort caused by the device. To achieve this, the device should be constructed from soft, biocompatible materials with low modulus, matching the mechanical properties of the surrounding tissues. Additionally, a thinner profile is necessary to reduce the risk of the user feeling the device during movements, ensuring long-term comfort and compliance [[241], [242], [243]].

**Fig. 7:**
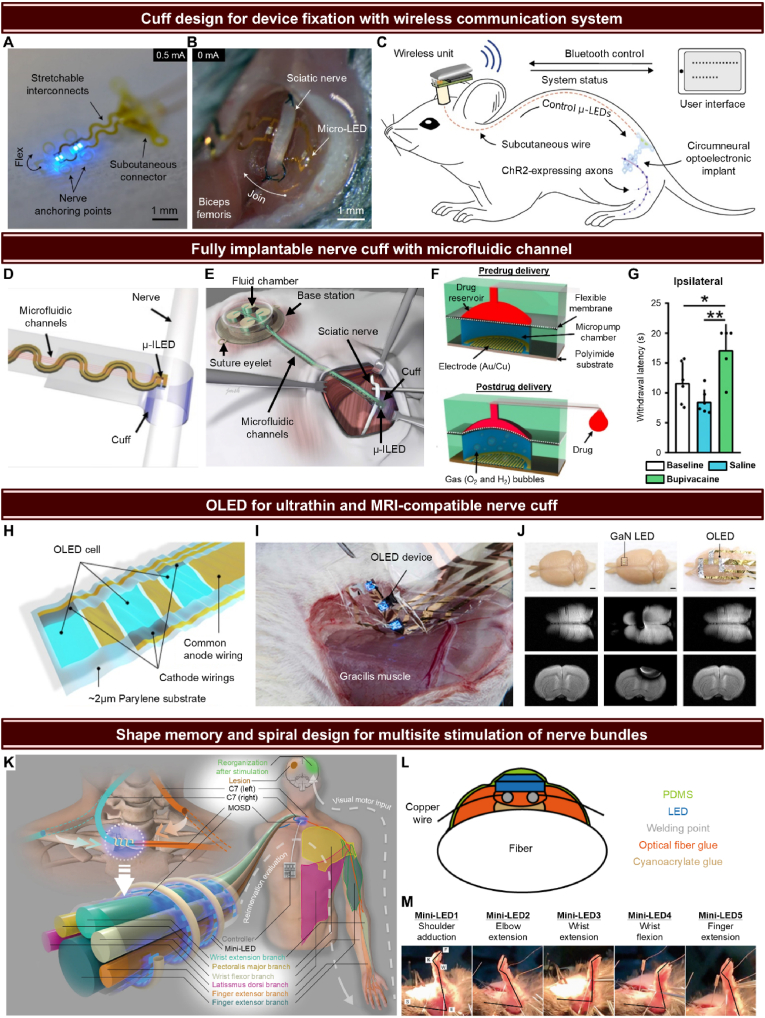
Various advanced optogenetic devices on somatic nervous systems. (A) Image of a flexible μ-LED array wrapping around the sciatic nerve, designed with stretchable interconnects that allow it to conform to the nerve's surface while accommodating movements, ensuring consistent and stable optical stimulation. (B) Anchoring points for secure fixation, showing how the μ-LED array can be sutured to the nerve to maintain precise positioning even during muscle contractions and movement, crucial for long-term stable neuromodulation. (C) Schematic of the wireless control system for free movement, depicting a Bluetooth-enabled unit that communicates with the subcutaneously implanted LED array, allowing real-time modulation of stimulation parameters in freely moving animals, ensuring natural behavior without physical tethering. (A to C) Reproduced from Ref. [146], with permission from Springer Nature. (D) Flexible and stretchable nerve cuff incorporating μ-LEDs and microfluidic channels, designed for dual functionality in optogenetic stimulation and localized drug delivery. (E) Schematic of the wireless, battery-free optofluidic system, illustrating the NFC-powered base station used to control μ-LED illumination and drug delivery, providing power and communication for untethered operation. (F) Mechanism of the ultralow-power electrochemical micropump system integrated within the cuff device, illustrating how the micropump operates via water electrolysis to generate oxygen and hydrogen gas, expanding a flexible membrane that applies pressure to the drug reservoir. (G) In vivo result showing the effect of bupivacaine delivery through the microfluidic cuff system, demonstrating how the system effectively blocks nerve signals by administering the local anesthetic. The graph measures paw withdrawal latency in response to a thermal stimulus applied to the treated side (ipsilateral), showing a significant increase in latency after drug delivery compared to the baseline and saline control groups, confirming the successful induction of a nerve block and a reduction in thermal sensitivity in freely moving mice. (D to G) From Ref. [142]. Reprinted with permission from AAAS. (H) Schematic image of an ultraflexible OLED applied to the sciatic nerve, fabricated on a 2-μm-thick Parylene substrate integrating three emission cells and two wirings. (I) Optical image of the OLED device applied to the sciatic nerve in an in vivo experiment, highlighting how the flexible OLED conforms to the curved surface of the nerve without causing mechanical damage, as shown by the absence of significant immune response or myelin sheath alteration. (J) MRI scan image comparing the OLED and a conventional GaN LED attached to the brain, showing minimal signal interference caused by the OLED, making it MRI-compatible, unlike the GaN LED that produces significant signal loss in the surrounding area. (scale bar, 2 mm) (H to J) [252] Copyright (2020) National Academy of Sciences. (K) Shape memory and spiral optoelectronic device applied to the C7 nerve bundle, showing its capacity for multisite stimulation by positioning multiple mini-LEDs along individual branches of the C7 nerve, allowing for selective neuromodulation of different motor functions. (L) Schematic of the spiral-shaped device, illustrating its construction using shape memory polymer (SMP) with integrated mini-LEDs, PDMS coating for flexibility and biocompatibility, and copper wiring for stable electrical connectivity. Welding points and optical fiber glue ensure the secure attachment and precise delivery of light to targeted nerve branches. (M) In vivo images showing the results of selective stimulation of individual nerve branches within the C7 bundle, where each mini-LED independently controls specific movements. The experiment successfully demonstrated shoulder adduction, elbow extension, wrist flexion, and finger extension using targeted light delivery to corresponding nerve branches. (K to M) Reproduced from Ref. [118], with permission from Springer Nature.

Nerve cuffs or conduits are widely used in neuroscience research due to their ability to conformally wrap around afferent and efferent nerves, making them ideal for applications like recording electrophysiological signals or providing electrical stimulation via integrated electrodes [[244], [245], [246], [247], [248]]. Recently, there has been growing interest in devices that incorporate LEDs to enable direct optical stimulation of nerves, advancing the field of optogenetics [142,238,239,249]. In the study by Michoud et al., a cuff-shaped, stretchable LED implant was developed for epineural optogenetic activation [53]. This custom-designed device is tailored to the dimensions of the sciatic nerve, allowing it to flexibly conform around the nerve, ensuring reliable contact for consistent stimulation (Fig. 7A). Anchoring points on both sides of the device allow for easy suturing after implantation (Fig. 7B). This design ensures the implant remains securely in place even in dynamic environments like the SNS, where constant muscle movement and body motions would otherwise displace it.

For optogenetic experiments targeting the SNS, as with other nervous systems, a wireless setup is essential to allow free movement in animals, ensuring natural behaviors are maintained without tethering, which could otherwise induce behavioral artifacts. The overall system designed by Michoud et al. combines the cuff-shaped implant with a subcutaneously placed wire connected to a head-mounted wireless control unit (Fig. 7C) [238]. This unit, controlled via Bluetooth, enables real-time modulation of light delivery to ChR2-expressing axons in freely moving animals. The wireless design minimizes physical restrictions, allowing long-term behavioral experiments to be conducted without interference, while the user interface offers precise control over stimulation parameters.

Another stretchable nerve cuff incorporating μ-LEDs was developed by Zhang et al., specifically designed for wireless, battery-free optogenetic and pharmacological modulation of peripheral nerves [142]. This cuff-shaped device features integrated μ-LEDs and microfluidic channels, enabling dual functionality for both light-based stimulation and localized drug delivery (Fig. 7D). The cuff is constructed from soft, biocompatible materials, including PDMS, ensuring that it conforms closely to the nerve, providing a secure fit while maintaining mechanical compliance. The device employs an NFC wireless module for both power harvesting and control. Power transfer is achieved via inductive coupling at a frequency of 13.56 MHz, which is commonly used in NFC-enabled consumer electronics [250]. The wireless operation is further enhanced by a small, lightweight base station that contains drug reservoirs and control electronics. The base station is implanted subcutaneously, and the system is entirely battery-free, which not only eliminates concerns about battery lifespan or recharging but also makes the device fully implantable (Fig. 7E).

The drug delivery mechanism of Zhang's system is powered by an ultralow-power electrochemical micropump (Fig. 7F) [142]. The micropump utilizes a water electrolysis process in a chamber containing an aqueous potassium hydroxide (KOH) solution. When a small voltage is applied to the interdigitated electrodes (made from Au/Cu), electrolysis produces oxygen and hydrogen gas bubbles. These gases expand within the micropump chamber, deforming a flexible membrane and applying pressure to the drug reservoir. As the membrane deflects, it forces the drug from the reservoir through the microfluidic channels and out of the cuff to the targeted nerve. In the in vivo drug delivery experiments, the device successfully modulated the thermal sensitivity of freely moving mice by administering bupivacaine, a local anesthetic. As demonstrated in Fig. 7G, the experiment measured the paw withdrawal latency in response to a thermal stimulus applied to the ipsilateral side (the side treated with the drug). After bupivacaine delivery, the withdrawal latency significantly increased compared to both the baseline and saline control groups, indicating that the drug effectively induced a nerve block and reduced the mice's sensitivity to heat.

When using organic-LEDs (OLEDs) instead of conventional inorganic LEDs, the mechanical advantages are significant, especially in biomedical applications where conformity to soft tissues is crucial. OLEDs are ultrathin, lightweight, and highly flexible, which reduces the mechanical stress on biological tissues compared to conventional inorganic LEDs [251]. Kim et al. developed an ultraflexible OLED device for optogenetics with remarkable mechanical properties (Fig. 7H) [252]. The OLED was fabricated on a 2-μm-thick Parylene substrate, providing both durability and flexibility. Each OLED cell consisted of an anode and cathode wiring on this thin substrate, with a total device thickness of under 3 μm. Immunological studies showed that the OLED device caused no significant immune response or mechanical damage to nerve tissues. In terms of optical performance, the OLED provided an optical power density of 0.5 mW/mm^2^, which exceeds the activation threshold (∼0.3 mW/mm^2^) required for channelrhodopsin-2 (ChR2) and other common opsins [253]. Kim et al. validated this in vivo by demonstrating optically induced neuronal activation and muscle contractions in ChR2-expressing transgenic animals. In several in vivo experiments, the OLED was applied to different peripheral nerves, including the gracilis muscle, sciatic nerve, and hindlimb skin (Fig. 7I). In these experiments, the OLED's flexibility enabled it to conform to the curved surfaces of the nerves and muscles, ensuring stable optical stimulation without causing physical harm. This highlights the OLED's versatility in targeting various nerve sites, underscoring its potential for broad applications in peripheral nerve stimulation. The OLED's materials, being non-magnetic and non-metallic, also offer critical advantages in MRI environments [254,255]. As shown in Fig. 7J, MRI scans revealed no signal interference or artifacts when the OLED device was placed on the brain, in contrast to conventional inorganic LEDs that often produce significant MRI artifacts. This MRI compatibility makes the OLED an ideal candidate for combined optical stimulation and imaging applications in neuroscience.

Some parts of the SNS include complex nerve bundles, such as the C7 nerve, which consists of multiple nerve branches that innervate different muscles [256]. Targeting individual branches within these bundles for precise neuromodulation poses significant challenges, as selective stimulation of each branch can result in more refined control over muscle movements. For example, selectively stimulating branches within the C7 bundle allows independent control of the shoulder, elbow, wrist, and fingers, which is crucial for applications in motor function restoration and neuromodulation. In addressing this challenge, Zheng et al. introduced an innovative device capable of delivering multisite optical stimulation to nerve bundles like C7 [118]. Their device features a spiral design that wraps around the nerve bundle, providing precise positioning of multiple mini-LEDs along the nerve (Fig. 7K). This design ensures that each nerve branch can be individually stimulated, offering more selective and controlled neuromodulation compared to traditional methods.

The key to this device's effectiveness lies in its construction (Fig. 7L). The device is made from a shape memory polymer (SMP), which allows it to initially be deployed in a straight configuration and then conform to the spiral shape once in place around the nerve [118,257]. This ensures secure contact with the nerve without the need for invasive fixation methods, while the integration of PDMS coating, copper wiring, and optical fiber glue enables the mini-LEDs to function effectively within the flexible and biocompatible structure, maintaining both electrical connectivity and mechanical stability even in dynamic environments. In vivo experiments demonstrate the device's success in stimulating individual nerve branches within the C7 bundle of a mouse (Fig. 7M). The device enabled selective stimulation of the shoulder, elbow, wrist, and fingers, which was not achievable with traditional electrical stimulation or single-site optical methods. This approach makes the device a valuable tool for applications where precise and targeted stimulation of nerve branches is needed, offering improved selectivity compared to existing methods.

## 4 Conclusion

Optogenetics has gained significant attention due to its high cell-type specificity and temporal resolution compared to traditional drug therapy and electrical stimulation. By integrating optogenetics with bio-implantable devices, it is now possible to selectively modulate various nervous systems, including the CNS and PNS. Given that each nervous system possesses distinct structural and mechanical properties, it is essential to fabricate devices according to specific design rules tailored to the unique requirements of each area.

In the CNS, encompassing both the brain and spinal cord, devices must be designed to accommodate unique anatomical and mechanical characteristics. For the brain, devices are either surface mounted to conform to the brain's surface or implanted as neural probes to reach deep brain regions. Surface mounted devices require thin, flexible structures that adapt to the brain's soft tissue, ensuring stable and consistent stimulation. Neural probes must be engineered to minimize insertion damage by considering the brain's softness and elasticity. The spinal cord, in contrast, exhibits more dynamic movements due to the flexibility of the spinal column. Devices for spinal cord modulation must, therefore, accommodate these movements by incorporating mechanical flexibility and stretchability to maintain stable operation. Effective delivery of stimulation to both superficial and deeper spinal regions is crucial, potentially requiring design considerations like using longer-wavelength light.

Devices targeting the ANS must endure the dynamic movements and significant stretchability of internal organs. Designs need to maintain stable contact under mechanical deformation, often requiring stretchable materials and structures that can accommodate organ expansion and contraction without compromising device integrity. In the SNS, devices must remain securely positioned despite dynamic body movements. Appropriate anchoring mechanisms or cuff-shaped designs are essential to minimize displacement due to movement and ensure consistent stimulation.

Overall, these design considerations ensure that bio-implantable optogenetic devices are optimized to meet the specific anatomical and functional demands of each nervous system region, enhancing their effectiveness and reliability. By adhering to these tailored design rules, advanced optogenetic devices can more precisely modulate neural activity, paving the way for new therapeutic options and expanding the capabilities of neuromodulation research.

Translating optogenetic technologies from rodent models and non-human primates to human clinical applications presents new challenges and opportunities. Moving from small animal models to humans necessitates the development of devices capable of covering larger anatomical areas while maintaining high spatial and temporal resolution. Designing scalable devices that can modulate extensive neural networks is essential. Achieving precise control over large cortical regions, for example, requires devices that deliver targeted stimulation across broad surfaces without compromising resolution.

Integrating multifunctionality into bio-implantable devices emerges as a pivotal direction for future development. Combining various signal measurements with multiple modulation modalities enables the capture of more precise and sophisticated neural signals, allowing for more complex neuromodulation strategies. This multifaceted approach can lead to a deeper understanding of neural networks and offer more effective therapeutic interventions. However, designing devices that can simultaneously perform sensing and stimulation across different modalities presents significant challenges, particularly in managing interference between these functions. Efficiently resolving interference is crucial to ensure accurate signal acquisition and effective neuromodulation without crosstalk or signal degradation.

Clinical applications demand additional considerations of biocompatibility, long-term stability, and safety. Materials used in device fabrication must be compatible with human tissue and function reliably over extended periods. The development of bioresorbable devices offers a promising direction, as they can perform their therapeutic function and then safely degrade within the body, eliminating the need for surgical removal and reducing the risk of long-term complications.

Advanced bio-implantable devices for human applications will enable more precise control over neural activity and improve the ability to modulate complex neural circuits. These advancements have the potential to revolutionize the treatment of neurological disorders and provide unprecedented insights into human functions. By bridging the gap between experimental models and clinical practice, optogenetics combined with advanced bio-implantable devices could significantly enhance the quality of life for patients with neurological conditions, offering new therapeutic options that were previously unattainable.

## CRediT authorship contribution statement

**Ju Young Lee:** Writing – original draft, Visualization, Validation, Conceptualization. **Taemin Kim:** Writing – original draft, Visualization, Validation, Conceptualization. **Shinil Cho:** Writing – original draft, Visualization, Validation, Conceptualization. **Jiho Shin:** Writing – review & editing, Validation. **Woon-Hong Yeo:** Writing – review & editing, Validation, Supervision, Funding acquisition. **Tae Soo Kim:** Writing – review & editing, Validation, Supervision, Conceptualization. **Ki Jun Yu:** Writing – review & editing, Validation, Supervision, Funding acquisition, Conceptualization.

## Ethics approval and consent to participate

Not applicable.

## Declaration of competing interest

The authors declare that they have no known competing financial interests or personal relationships that could have appeared to influence the work reported in this paper.
